# Multi‐Mode Mechanochromic Responses from Cholesteric Liquid Crystal Elastomer Tubes of Uniform Sheath

**DOI:** 10.1002/adma.202504461

**Published:** 2025-06-10

**Authors:** Jong Bin Kim, Shangsong Li, Kun‐Yu Wang, Yinding Chi, Shu Yang

**Affiliations:** ^1^ Department of Materials Science and Engineering University of Pennsylvania 3231 Walnut Street Philadelphia PA 19104 USA

**Keywords:** cholesteric liquid crystal elastomers, displays, mechanochromism, structural colors, tube

## Abstract

Materials that exhibit varied optical responses to different modes of mechanical stimuli are attractive for complex sensing and adaptive functionalities. However, most mechanochromic materials are fabricated from films or fibers with limited actuation modes. Here, hollow tubes of a symmetric sheath are created using cholesteric liquid crystal elastomers (CLCEs) at the sub‐millimeter scale. The oligomeric precursor is sheared in an elastomeric microchannel to form uniform thickness, overcoming gravity effect and Plateau‐Rayleigh instability. In addition, the coloration is achieved to be faster and have higher reflectivity compared to that of solid fibers. The tube can undergo axial, circumferential, and radial strains upon extension and inflation. The combination of molecular anisotropy and geometry of the tube enables highly sensitive mechanochromic responses in both azimuthal and axial directions: inflation causes red‐to‐violet shift (≈220 nm) at a circumferential strain of 0.57. The inflation of a bent tube generates another mechanochromic mode with a higher sensitivity to strain. Finally, display of 26 alphabets is achieved using 5 tubes, of which the positions can be reconfigured, and curvature‐dependent 3D photonic skins are demonstrated from tubes wrapped around 3D objects. The multi‐mode mechanochromic tubes will find applications for soft robotics, adaptive displays, wearable sensors, and spectrometers.

## Graphical abstract

The structural‐colored tube is created from cholesteric liquid crystal elastomers, exhibiting uniform color changes with high strain sensitivity upon extension and inflation. The color change occurs across multiple mechanochromic modes, highlighting the influence of molecular anisotropy and tubular geometry on strain sensitivity. The tubes demonstrate reconfigurable alphabet displays and 3D photonic skins.

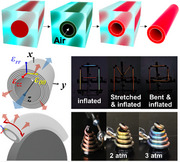

## 1 Introduction

Structural colors (SCs) reflected from periodically layered structures hold promise in sensing,^[^ ^1^, ^2^ ^]^ color displays,^[^ ^3^, ^4^ ^]^ and camouflage^[^ ^5^, ^6^ ^]^ in response to external stimuli, including mechanical force,^[^ ^5^, ^7^ ^]^ heat,^[^ ^8^, ^9^ ^]^ and solvent or humidity.^[^ ^10^, ^11^ ^]^ In particular, mechanical forces are attractive for fast (on the order of milliseconds) and broadband SC changes,^[^ ^5^ ^]^ for example, by uniaxial/biaxial stretching,^[^ ^12^, ^13^ ^]^ compression,^[^ ^14^ ^]^ or bending^[^ ^15^ ^]^ of soft materials. Patterning of elastomers or shape memory polymers followed by local deformation could convey information for visual recognition aided by techniques including electric field‐induced expansion of dielectric elastomers (DEs),^[^ ^6^ ^]^ stamping/indentation,^[^ ^16^, ^17^ ^]^ and pneumatic inflation.^[^ ^5^ ^]^ However, a large strain (> 50%) is often needed to achieve a noticeable color shift. The recoverability of the deformed patterns to the original state is dependent on the material elasticity and pattern size,^[^ ^17^ ^]^ typically on the order of tens of microns so that elastic energy is dominant over adhesion energy.^[^ ^18^ ^]^ Meanwhile, materials capable of multi‐mode mechanochromic responsiveness—where optical responses vary depending on the magnitude and type of the mechanical input—will be highly attractive. Nature does this by combining different optical components. For example, cephalopod skin is embedded with chromatophores, iridophores that act as Bragg reflectors, leucophores that scatter light, and papilla that change texture under pressure.^[^ ^19^ ^]^ Researchers have demonstrated multi‐mode optical responses by coupling mechanical stimuli with light, heat, and microfluidics,^[^ ^20^, ^21^, ^22^ ^]^ which, however, adds complexity to fabrication and manipulation. It will be ideal to achieve multi‐mode mechanochromic responses from a single material.

A broadband and pixelated display has been demonstrated in pneumatically‐inflatable cholesteric liquid crystal elastomer (CLCE) thin membranes, where color changes from near‐infrared (NIR) to ultraviolet (UV) with < 20% equi‐biaxial transverse strains.^[^ ^5^ ^]^ CLCEs are soft networks composed of consecutive rotating layers of aligned liquid crystal (LC) molecules. The native colors are determined by the helical pitch (*P*) with the reflection peak wavelength proportional to the pitch. They are promising candidates for mechanochromic response due to their highly tunable softness and high strain anisotropy.^[^ ^2^, ^23^, ^24^, ^25^, ^26^ ^]^ Nevertheless, thin membranes, confined on a substrate, have limited spatial reconfiguration and color display in 3D. CLCE fibers that can be knitted,^[^ ^27^ ^]^ knotted,^[^ ^28^ ^]^ or woven into fabrics^[^ ^29^, ^30^ ^]^ demonstrate a higher degree of configurational freedom. However, their color changes are typically realized by uniaxial stretching, which is essentially 1D. Moreover, a large uniaxial strain (> 100%) is necessary to shift from red to blue, which not only decreases color intensity but causes fibers to be easily fractured. Inflatable hollow CLCE fibers printed by core‐shell direct ink writing offer reconfigurable color changes in radial, axial, and circumferential directions.^[^ ^31^ ^]^ However, the shell thickness is asymmetric due to gravitational effects during fabrication, leading to inconsistent colors along the circumferential direction. Their sizes are limited to the millimeter‐scale because the precursor can spread after extrusion.

Here, we fabricate CLCE hollow tubes featuring a uniform sheath thickness in both azimuthal and axial directions. The tube can be stretched and compressed in different directions regardless of the tube's configuration, providing multiple pathways for 3D reconfigurable mechanochromic responses. To create the tube, we fill an elastomeric microchannel (diameter, *D*, 640, 910, and 1350 µm) with highly viscous CLCE precursors, followed by air injection, where the precursors experience evenly distributed shear force while effectively delaying the interfacial instability to form a uniform air core. Compared with isotropic liquids, the anisotropic CLC precursors are more resistant to shear deformation, where the Oseen‐Frank elastic energy for distortion makes the rearrangement of CLC molecules anchored at the channel interface thermodynamically unfavorable.^[^ ^32^, ^33^, ^34^ ^]^ The fabricated CLCE tubes are more strain sensitive than fibers: it requires 0.48 axial strain for tubes versus 0.58 for fibers to achieve a 15% shift in the reflectance peak wavelength. Upon inflation, the circumferential strain required to achieve the same degree of peak shift is reduced to 0.25. The anomalous mechanical responses to extension and inflation could be ascribed to the anisotropic nature and the soft elasticity of CLCEs.^[^ ^35^, ^36^, ^37^ ^]^ Further, the degree of color change upon inflation is influenced by the curvature of the substrate that the tube adheres to.

## 2 Results and Discussion

### 2.1 Fabrication of the CLCE Tubes and Control of the Structural Coloration

To create the uniform cylindrical tubes, we first fill the elastomer microchannel, made from polydimethylsiloxane (PDMS), with a highly viscous cholesteric liquid crystal (CLC) precursor mixed with oligomers, monomers and mesogenic solvents, followed by air injection to form a uniform air core. The interface between air and precursor is susceptible to deformation driven by surface tension, which minimizes energy by favoring a spherical configuration, thereby causing instability. The density contrast between the precursor and air at the interface could lead to gravitational effects, causing asymmetric CLCE distribution within the tube. To delay the instability and achieve axial and azimuthal uniformity, high viscosity of the CLCE precursor is preferred, which, however, could also cause non‐uniform formation of the CLC phase at the interface. Here, we formulate the CLCE precursor using a mixture of LC monomers, CLC oligomers together with the mesogenic solvent to both achieve a high viscosity (600 Pa·s) and enable the rapid formation of the cholesteric phase (Figure S1a, Supporting Information; see the Experimental Section for details on the precursor preparation).^[^ ^31^ ^]^

When the CLCE precursor fills the PDMS microchannel, the self‐assembly of the cholesteric phase begins at the PDMS‐precursor interface. Prior to the full development of structural color, air is injected into the microchannel to shape the precursor into a sheath configuration. Since the channel is elastic and an air core is created after depressurization, our system bridges the classical Bretherton model^[^ ^38^, ^39^ ^]^ and the airway reopening model in bronchi.^[^ ^40^, ^41^ ^]^ In the classical Bretherton model, a long air bubble propagates through a rigid, liquid‐filled capillary, leaving behind a thin liquid film along the wall. This model describes the interplay between viscous stresses and surface tension, and determines the steady‐state film thickness. Conversely, the airway reopening model examines the air injection into a fluid‐filled elastic channel. It explores how pressure affects the speed of air flow, which inflates the channel but does not leave an air core after depressurization when the capillary number *Ca* = *µU*/*γ* is larger than 0.5,^[^ ^40^, ^42^ ^]^ where *µ* is the viscosity, *γ* is the surface tension, and *U* is the air front speed. Here, the elastic PDMS is chosen to create microchannels because bright and uniform colors can be formed on PDMS.^[^ ^31^ ^]^ Upon air injection, a uniform air core is formed after depressurization when 4 < *Ca* < 35; here, *γ* = 35 mN m^−1^ for the CLC precursors (Figure S1b, Supporting Information). The discrepancy with airway reopening is attributed to the high pressure generated by the oligomer's high viscosity, sub‐millimeter scale of the channel, and the high longitudinal wall tension due to the large thickness of PDMS walls (≈5 mm).

**Figure 1:**
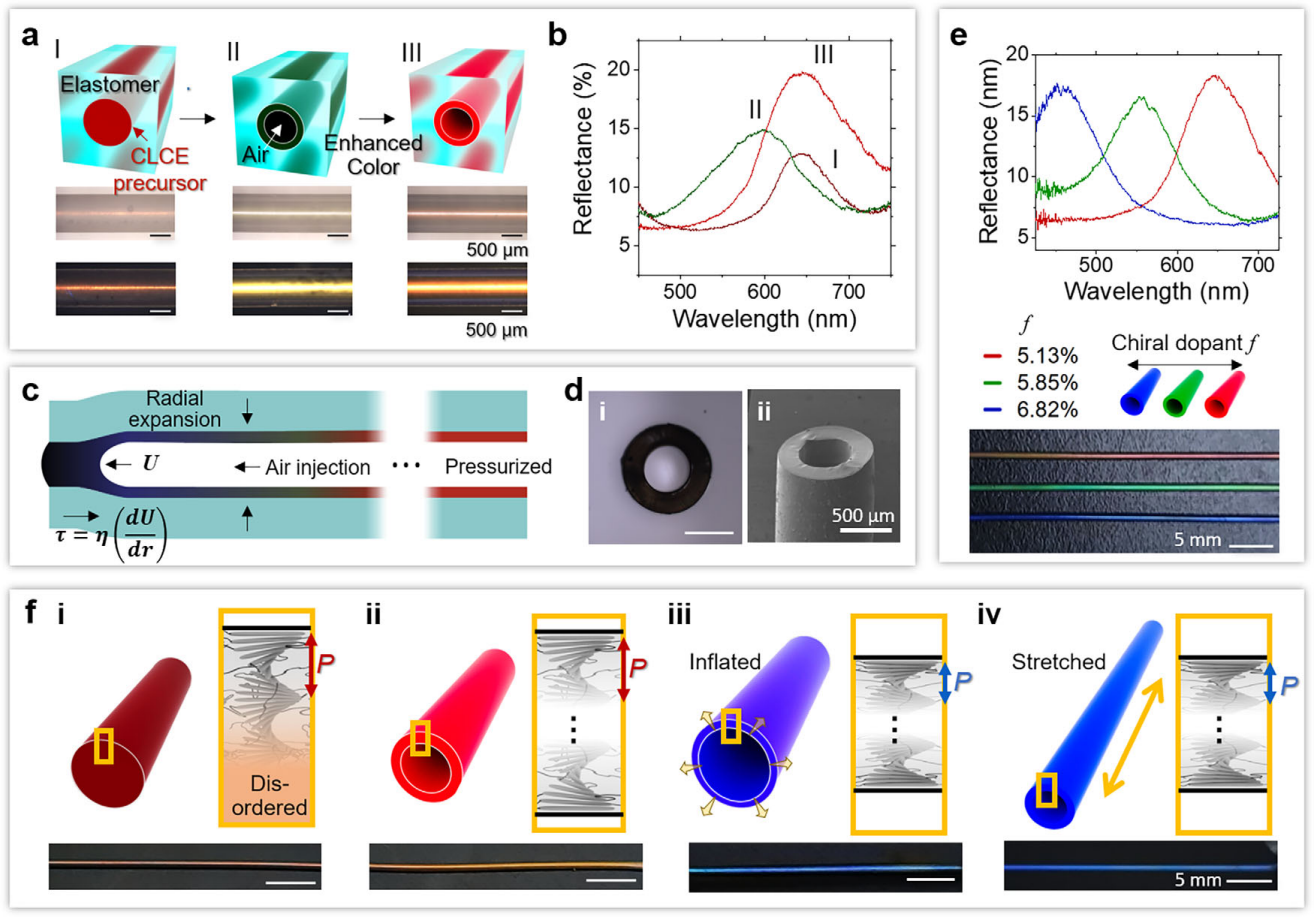
Mechanochromic CLCE tubes. a) Schematic illustration of the fabrication of a tubular CLCE (1st row), the corresponding reflective optical microscopy (OM) images (2nd row), and polarized optical microscopy (POM) images (3rd row). I, 30 min after filling the channel with the CLCE precursor. II, immediately after air injection in I. III, 1 min after II. b) Reflectance peaks from the CLCE tubes at three states indicated in a. c) Cross‐sectional view of an elastic channel with air injection. d) Cross‐sectional OM image i) and scanning electron microscopy (SEM) image of the CLCE tube. e) Reflectance spectra (top) and the corresponding photos (bottom) of the red, green, and blue CLCE tubes with varying chiral dopant concentrations (wt.%). f) Schematics of the alignment of CLC molecules in a fiber i), tube ii), tube upon inflation iii) and stretching iv), and the corresponding colors.

The air injection pressure (1.5–2 atm) causes a blueshift in the annular precursor's color (**Figure** **1**a,b). The radial inflation of the PDMS microchannel induces radial compression and shear force in the CLC sheath (Figure 1c), which decreases the cholesteric pitch, even without crosslinking (Figure S2, Supporting Information). 1 min after air injection, the stress recovers, and the reflectance peak gradually redshifts, returning to its original position. Meanwhile, a color that is more intense than that obtained in the fiber is observed (Figure 1b). The resulting air core has a circular cross‐section as the PDMS channel radius (320, 455, and 675 µm, respectively) is smaller than the capillary length $l=\sqrt{\gamma /\rho g}$ = 1.63 mm, where *ρ* is the density of the CLCE precursor and *g* is the acceleration of gravity (Figure 1d(i)). The tube thickness is uniform in the azimuthal direction due to the evenly distributed viscous forces by the advancing air finger within the precursor (Figure 1d(ii)), which is essential to achieve the uniform color changes upon later stretching or inflating the formed CLCE tube.

The pitch of CLCE can be tuned by the concentration of the chiral dopant. A higher concentration facilitates the rotation of LC molecules within the cholesteric phase, leading to a smaller pitch and blueshift of the color (Figure 1e). At the same amount of the chiral dopant, the CLCE fiber and tube have the same reflection peak wavelength. However, the tube shows 154% higher reflection intensity (Figure 1b), indicating enhanced CLC alignment. We hypothesize that color enhancement comes from the geometrical differences between fibers and tubes, and the result of air injection. In a PDMS microchannel filled with CLC precursors, since the CLC assembly occurs at the precursor‐PDMS interface, CLC phase inside the core becomes less ordered when moving away from PDMS due to diminishing anchoring force (Figure 1f(i)).^[^ ^43^ ^]^ When air is injected, the color is initially dimmed and blue‐shifted due to the disturbance by radial expansion. However, the axial shearing force promotes cholesteric phase assembly.^[^ ^3^ ^]^ In addition, a new precursor‐air interface is formed, which accelerates the formation of CLC phase.^[^ ^44^ ^]^ Therefore, the color at the sheath not only restores quickly but becomes brighter than before air injection. The effect of shear forces induced by air injection, and the resulting enhanced alignment of the CLC phase, are further supported by the optical microscopy (OM) images observed in the reflection mode (Figure S3, Supporting Information). Pointy defects are observed from the fiber, whereas the tube shows grains elongated along the shear direction with brighter coloration and fewer defects. It is worth noting that the CLC phase is formed vertically to the tube surface, albeit the shear force during the air injection, as confirmed by the same extent of blueshift at the same but opposite angles of specular reflection (Section S1 and Figure S3a,b, Supporting Information). The tube with enhanced color can shift its color through inflation or extension, which compresses the helical pitch and results in a blueshift (Figure 1f(iii,iv)). The vertical orientation of the helical pitch is further confirmed when the tube sheath is thinned (Section S1 and Figure S3c,d, Supporting Information). A more detailed discussion of the color‐changing deformation will be presented later.

**Figure 2:**
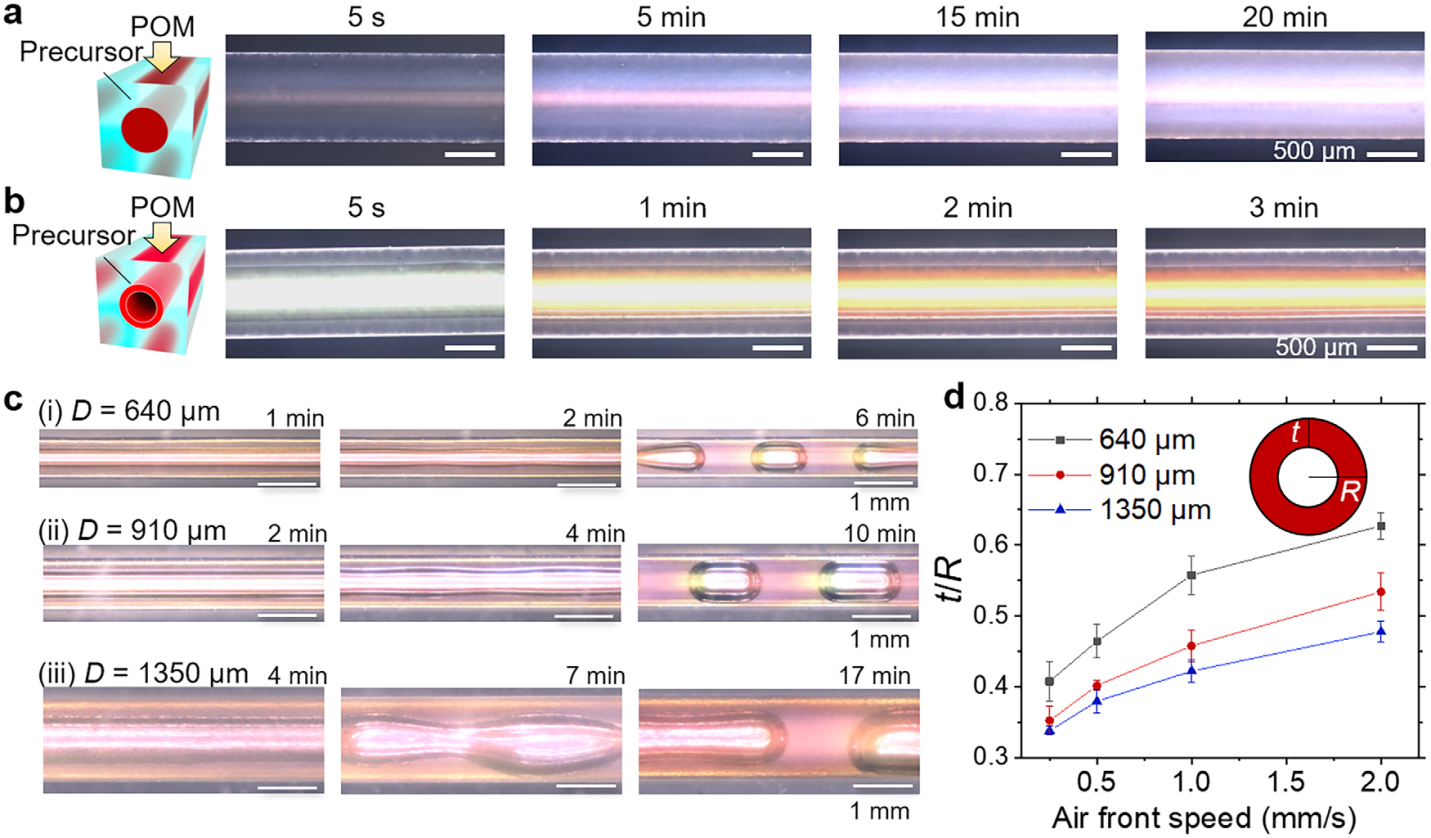
Control of the tubular geometries and colors in CLCE tubes. a,b) Time‐lapse POM images at different waiting periods after filling the CLCE precursor into the PDMS channel (a) and after injecting air, which is performed 2 min after the precursor fills the channel (b). c) Time‐lapse reflective OM images after air injection into the PDMS channels with variable diameters, *D*. d) *t*/*R* versus air front speed from various channel sizes (*n* = 4).

For color displays, it is important to ensure uniform color changes while maintaining high brightness. However, it is difficult to achieve both aims due to the trade‐off between the viscoelastic properties of precursors and gravity effects. While viscoelasticity mitigates the impact of gravity, enabling the formation of relatively uniform sheath structures, it also hinders the self‐assembly dynamics essential for achieving uniform SCs with high intensity. This challenge becomes even more profound as the size decreases to the sub‐millimeter scale. Here, we analyze the effects of air propagation and the subsequent time‐dependent change of SCs at the CLC‐precursor sheath in a microchannel. In a fibrous geometry with *D* of 910 µm, it takes ≈15 min to reach the maximal degree of the CLC phase formation (see polarized optical microscopy, POM, images shown in **Figure** **2**a).^[^ ^10^ ^]^ However, when air is injected 2 min after the precursor fills the channel, the time required for full coloration is reduced to less than 2 min (Figure 2b).^[^ ^43^ ^]^ The required time remains unchanged even when air is injected after the full coloration (Figure S5, Supporting Information). Therefore, waiting for complete coloration of the precursor in a fibrous geometry is not strictly necessary, and fully color‐developed tubes can be fabricated even faster than fibers.

A uniform tube thickness is also essential to achieve a consistent mechanochromic change along the axial direction. However, the CLC precursor shows higher loss moduli than the storage moduli across all angular frequencies (Figure S6, Supporting Information), indicating the dominance of a viscous behavior. Thus, surface‐tension‐driven instabilities are more likely to occur, even though the high viscosity (600 Pa·s) of the precursor helps to maintain the sheath longer (Figure 2c). As the channel size decreases, curvature and surface tension effects become more severe due to higher Laplace pressure. Precursor sheaths in a tube with *D* = 910 and 1350 µm become unstable after 4 and 7 min, respectively, whereas that with *D* = 640 µm only takes 2 min. Given that coloration in tubular structures is improved 1 min after air injection (Figure 1b), and it is shorter than the onset of instability, we UV cure the precursor after 1 min unless otherwise specified.

After confirming that CLCE tubes can achieve uniform thickness and colors, we investigate the factors influencing their thickness. Bretherton's problem reports the relationship between the thickness of the tube wall and *Ca* in a rigid channel.^[^ ^38^, ^45^ ^]^ While the airflow in the classical Bretherton model is driven solely by the Laplace pressure at the air front, that in elastic channels includes the pressure formed by viscous and inertial forces. In addition, the elastic modulus of the channel and viscoelasticity of the precursors make it difficult to predict tube thickness analytically or numerically.^[^ ^41^ ^]^ While existing theoretical models, such as the extended Bretherton model, are not applicable in the high‐*Ca* regime of our system (*Ca* > 4), our experimental results indicate that the film thickness still varies systematically with the air front speed (*U*), the sole variable determining the capillary number (*Ca* = *µU*/*γ*) under our experimental conditions (Figure 2d). The sheath thickness increases with the air front speed (see summary in **Table** **1**), as expected from Bretherton's model. The uniform thickness along both the axial and azimuthal directions was confirmed in CLCE tubes with length up to 5 cm (Figures S7 and S8, Supporting Information; see *Experimental section*). When the tube becomes longer, the pressure difference between the initial and final air injection stages leads to significant variations in the degree of channel inflation, thus hindering uniformity of the tube diameter. Unlike Bretherton's problem, changing channel diameters produces different *t*/*R* profiles as a function of air front speed, where *t* is a sheath thickness and *R* is a channel radius. Smaller channels create thicker tubes, because the pressure from increased viscous resistance broadens the cross‐section, leaving more precursor on the PDMS wall.

**Table 1:** Sheath thickness as a function of air injection speed and channel diameter.

Outer diameter	Air injection speed			
	0.25 mm s^−1^ [µm]	0.5 mm s^−1^ [µm]	1 mm s^−1^ [µm]	2 mm s^−1^ [µm]
640 µm	130.4 ± 9.1	148.6 ± 7.6	178.4 ± 8.8	200.7 ± 6.1
910 µm	160.3 ± 9.2	182.8 ± 3.0	208.3 ± 10.4	243.0 ± 12.0
1350 µm	228.1 ± 4.3	256.2 ± 11.0	284.9 ± 10.8	322.5 ± 10.0

### 2.2 Mechanochromic Modes of the CLCE Tubes

**Figure 3:**
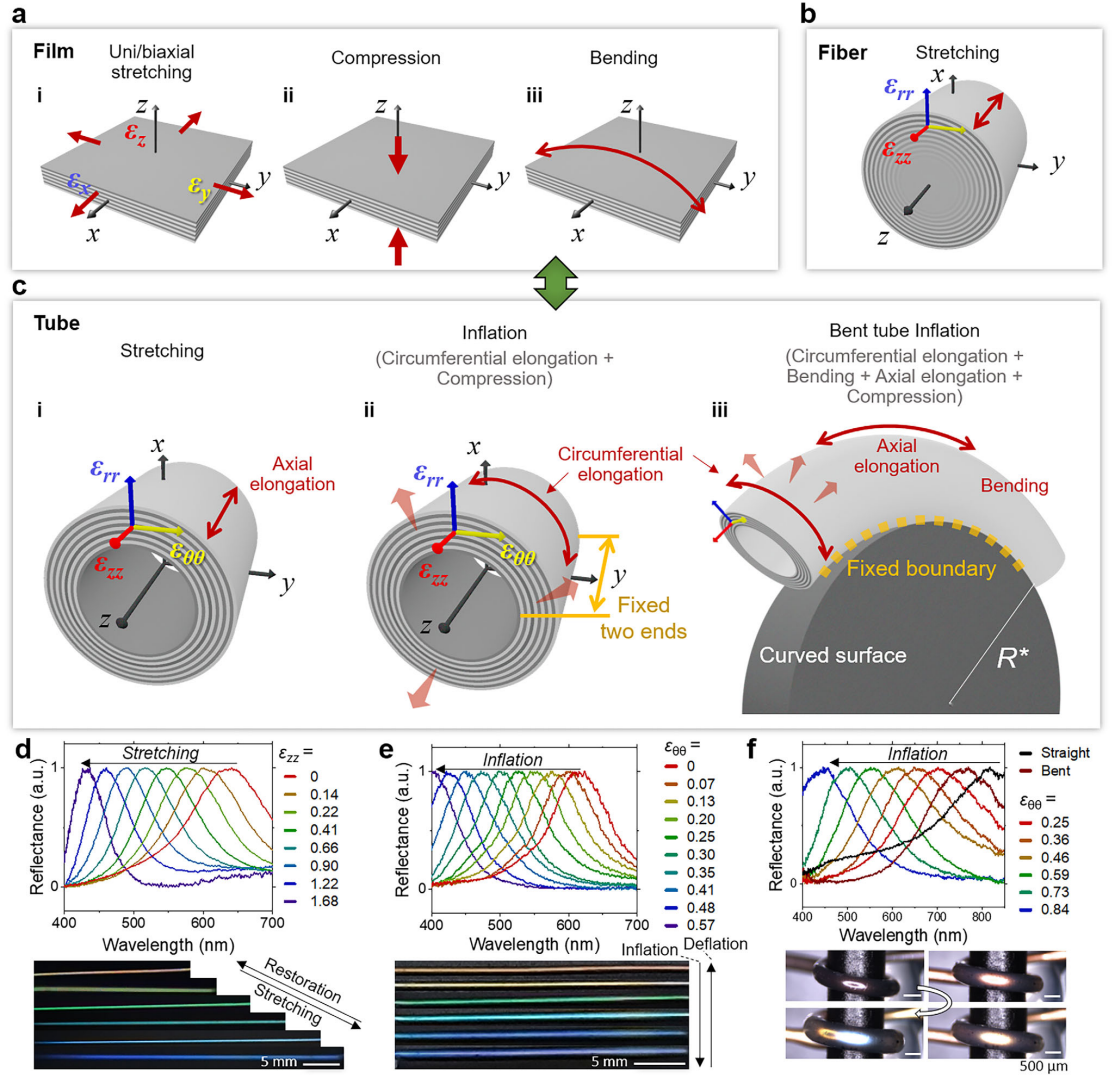
Mechanochromic modes of a CLCE film, a fiber, and a tube. a) Schematic diagrams of the three modes of mechanical deformation in a CLCE film. b) Schematic diagram of a CLCE fiber under stretching. c) Schematic diagrams of a CLCE tube under stretching, inflation, and bent tube inflation. d–f) Reflectance spectra and the corresponding photos of the CLCE tubes under deformation corresponding to c.

The successful fabrication of the CLCE tubes of uniform thickness enables us to investigate the multi‐mode mechanochromic responses by stretching and inflation. There are three modes of mechanical deformation: uni‐ and biaxial stretching, compression, and bending (**Figure** **3**a). Under uniaxial stretching, a film is thinned, which is governed by Poisson's ratio ν as ɛ_z_ =  νɛ_*x*_ or ɛ_z_ =  νɛ_*y*_. Here, ɛ_z_ is the strain in the thickness direction, ɛ_x_ and ɛ_y_ are the strains in the in‐plane directions, and ν ≤ 0.5 (Figure 3a(i)). The strain sensitivity of this thinning is lower than that of compression, which directly influences ɛ_z_ and change of the peak wavelength, Δ*λ*, which is the difference between the peak wavelength before and after applying strain. Δ*λ* ∝(*t*_1_ − *t*_0_)/*t*_0_, where *t*_0_ and *t*_1_ are the initial film thickness and that after compression, respectively (Figure 3a(ii)). The color change induced by a bending strain *ɛ_xx_*, which is determined by the curvature, can be expressed as Δ*λ*/*λɛ_xx_* t remains nearly constant when ɛ*_xx_* < 0.4 (Figure 3a(iii)).^[^ ^15^ ^]^ Then, the direction that induces color changes in a fiber is 1D, along the axial direction; the radial strain is ɛ_rr_ =   − ν_zr_ɛ_zz_, where *ɛ*_rr_ and *ɛ*_zz_ are radial and axial strains, and ν_*zr*_ is the Poisson's ratio showing the change of the radial strain to the axial strain (Figure 3b; see Section S2, Supporting Information).

The stretching and inflation of the tube induce three strain components, including ɛ_rr_, ɛ_θθ_, and ɛ_zz_ (Figure 3c(i,ii)). Stretching a tube made from an isotropic material can be described in the same way as stretching a fiber (see Section S2, Supporting Information). When increasing the pressure (*p*) to inflate a tube with two free ends from 1 to 2.78 atm, the tube is shortened in the axial direction, shifting the reflectance peak from 824 to 548 nm. When *p* is increased to 3.47 atm, the tube elongates, shifting the reflectance peak from 548 to 460 nm (Figure S9, Supporting Information). This phenomenon differs from that of an elastomer tube with an isotropic polymer arrangement, which elongates during inflation.^[^ ^46^ ^]^ It can be attributed to the soft elasticity, which describes the stress‐free deformation of LCEs in the direction perpendicular to the LC director due to the molecular anisotropy. In a tube, we suppose that directional injection of the CLC precursor, combined with shear forces from air finger propagation leaves a residual pseudo‐nematic phase in the axial direction. It is evidenced by the presence of nematic‐isotropic transition temperature at 103 °C and the axial shrinkage of the CLCE tubes placed on a hot plate (≈110 °C) (Figure S10, Supporting Information). It makes the tube more compliant circumferentially than axially,^[^ ^37^ ^]^ despite the formation of cholesteric phases near the tube walls,^[^ ^47^ ^]^ which leads to axial shortening during tube inflation. Namely, the tensile circumferential strain induced by tube inflation, in accordance with Poisson's effect, results in two compressive strains along axial and radial directions. Therefore, anchoring both ends of the tube lets compression solely occur in the radial direction, which further enhances the color change sensitivity. The tubes with both ends fixed remain straight while being inflated with a full visible spectrum change, eliminating the need to adjust the end positions to prevent bending due to elongation. Also, the pressure difference between the inner and outer side of the CLCE tubes results in the radial compression that enhances the sensitivity. During both elongation and inflation of the tube, the thickness uniformity is preserved, and the color change remains reversible (Figures S11–S13, Supporting Information).

Furthermore, the flexibility of the tubular geometry allows for tube extension in 3D through inflation while the tube is bent (Figure 3c(iii)). It results in simultaneous axial elongation, circumferential elongation, and eventually radial compression, which enhances strain‐induced color sensitivity compared to any single actuation mode. In each mode of deformation, we examine the lengths and outer diameters of the tubes and correlate each type of strain with the color change, where Δ*λ*/*λ*_0_ is presumed to be |ɛ_*rr*_|.^[^ ^5^ ^]^ For inflation, the outer diameter is considered to calculate ɛ_θθ_, as color primarily originates from the outer wall. The three different modes discussed so far exhibit varying sensitivities of color change to strain, due to differences in configuration and boundary conditions. The peak shift of ≈220 nm from the initial state is attained at ɛ_zz_ = 1.68, ɛ_θθ_ = 0.57, and ɛ_θθ_ = 0.46 for stretching, inflation, and bent‐inflation, respectively, as shown in the reflectance spectra (Figure 3d–f). This confirms that tube inflation requires less strain in the circumferential direction than in the axial extension to achieve the same peak shift, and even less when the tube is bent. Stretching and inflation of the tube exhibit distinct trends in reflectance intensity measured within a 25 µm‐diameter region (Figure S14, Supporting Information). Stretching follows the same behavior reported for CLCE fibers:^[^ ^29^, ^30^ ^]^ the intensity initially increases as distortion causes the breakdown of circular polarization, affecting both the right‐ and left‐handed components and enhancing reflection,^[^ ^48^ ^]^ and then decreases as uniaxial strain unwinds the helices, resulting in a loss of chiral photonic selectivity.^[^ ^13^ ^]^ In contrast, the CLCE tube maintains relatively consistent reflectance intensity upon inflation, as the inflation flattens the tube surface and the fixed axial strain alleviates the loss of chiral selectivity.

### 2.3 Strain Sensitivity Governed by Geometric Constraints in Different Modes of Tube Deformation

Both the extension and inflation of the tubes can be influenced by geometric factors. The axial strain during inflation is dependent on whether the ends are fixed, and extension is accompanied with a reduced inner volume, causing additional radial stress. Such deformation deviates from the Poisson's ratio υ that defines the relationship between the two orthogonal strains under a uniaxial stress. Therefore, to compare the effect of different types of mechanical deformation to the CLCE tubes, we quantify the sensitivity of the target strain change in response to the applied strain as *N*_ij_ (i, j = θ, r, z) = − ln (1 + ɛ_ii_)/ln (1 + ɛ_jj_).

**Figure 4:**
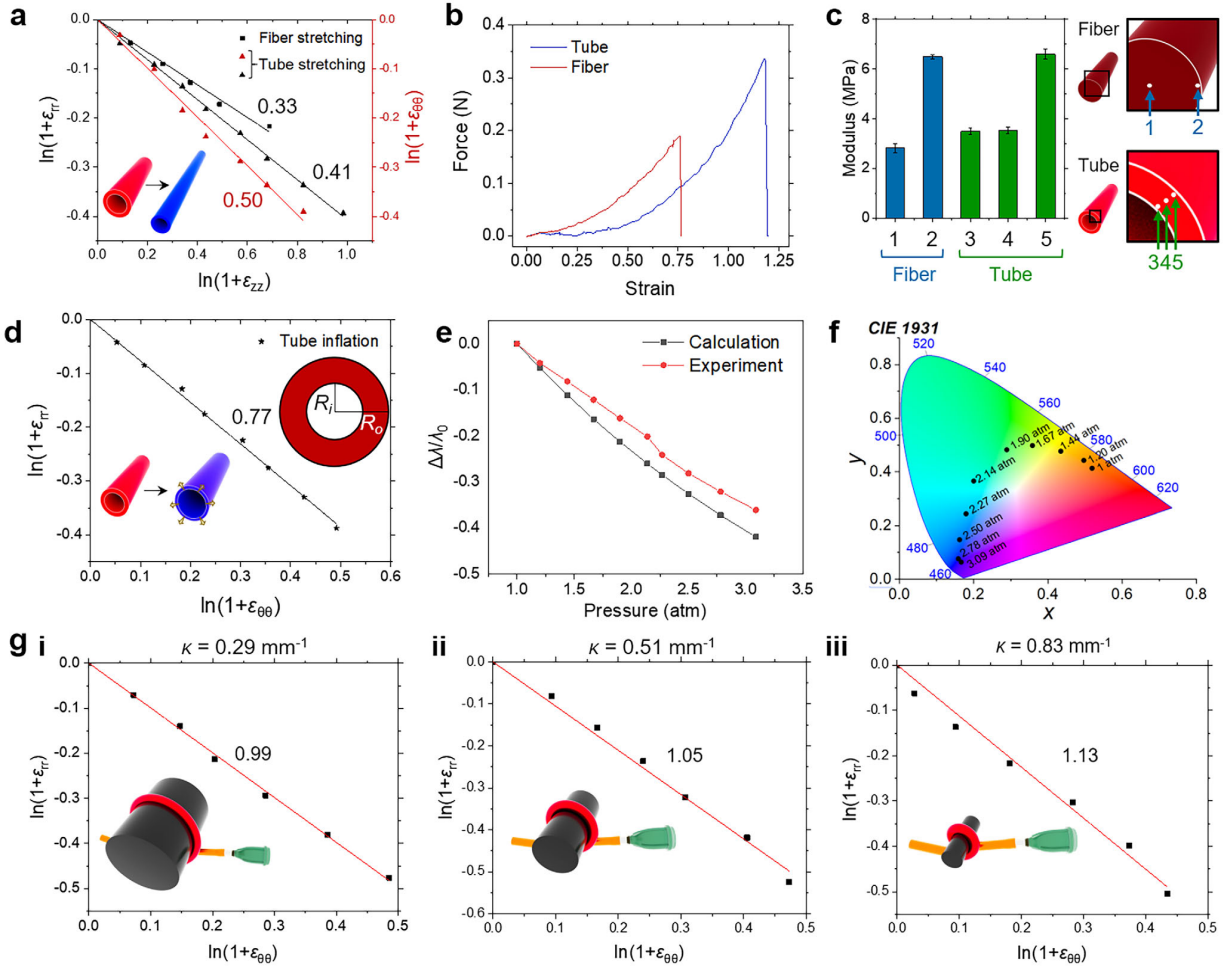
Analyses of three color‐changing modes of the CLCE tubes. a) Strain relationship when the CLCE fiber and tube are under axial stretching. b) Force‐strain relationship of a CLCE fiber and a CLCE tube. c) Indentation moduli at various locations on the cross‐section of the CLCE fiber and tube, respectively, measured by AFM. d) Strain relationship of the CLCE tube upon inflation. e) Relationship between the wavelength peak shift and pneumatic pressure. f) CIE diagram according to the pneumatic pressure that inflates the CLCE tube. g) Strain relationship of bent CLCE tubes wrapped on cylinders of different curvatures upon inflation.

First, upon stretching, tubes exhibit higher strain sensitivity than fibers; *ν* of fibers is 0.33, while *N*_zr_ and *N*_zθ_ for tubes are 0.41 and 0.50, respectively (**Figure** **4**a). Tubes also show a higher fracture strain, 1.18, compared to 0.75 for fibers (Figure 4b). It originates from the formation of a pseudo‐nematic phase formed by air injection, strengthening the mechanical properties along the longitudinal direction. Therefore, it allows a broader range of color shifts (Figure 4b). This fact makes tubes more advantageous in terms of both the range and sensitivity of color changes. The increased strain sensitivity is attributed to the stiffness difference between the cholesteric layers at the tube's outer wall (stiffer) and the pseudo‐nematic layers inside (softer), as well as to the compression of the tube wall during stretching. The stiffness contrast is confirmed by atomic force microscope (AFM) indentation tests (Figure 4c). While the outer walls of the fiber and the tube have comparable elastic moduli, the inner portion of the tube is stiffer than the fiber's core, suggesting molecular rearrangement in the large fiber core, which reduces the degree of color change. In contrast, tubes have a smaller cross‐sectional area and greater stiffness in their inner portion, limiting molecular displacement in the cholesteric phase. When compressed, the inner volume enclosed by two clips at the tube's ends increases to 9% at ɛ < 0.2, followed by a decrease to ‐21% when 0.2 < ɛ < 0.5 (Figure S15, Supporting Information). The initial surge in volume upon tube stretching at ɛ < 0.2 can be explained theoretically as:

(1) $$\Delta V=\pi R_{i}^{2}L\left(e^{\varepsilon_{zz}\left(1-2\nu_{rz}\right)}-1\right)$$

which is positive since 0 < *ν*_rz_ < 0.5 (see Section S3, Supporting Information).^[^ ^49^ ^]^ However, as the strain increases from 0.2 to 0.5, stress increases slowly while the inner volume decreases, suggesting a possible transition from polydomain to monodomain state (Figure 4b; Figure S15, Supporting Information). Therefore, the circumferential direction of the tube with nematic order is more compliant than its axial direction,^[^ ^37^ ^]^ making the color change more susceptible to the strain during tube extension. Simulations of the stretching of the tube and the fiber made from isotropic materials reveal identical, position‐independent strains across the stretched cross‐sections, supporting the critical role of CLCE anisotropy (Figure S16, Supporting Information).

Next, we quantify the strain‐induced color sensitivity upon tube inflation by *N*_θr_ when ɛ_zz_ = 0 due to the fixed boundary condition. When tube is inflated, *N*_θr_ = 0.77 due to the combination of circumferential expansion and compression of the tube wall (Figure 4d). The strain relationship during tube inflation is derived from a thick‐wall model with axisymmetric stress and negligible boundary effect as *L* > *R*_o_, where *L* is the tube length and *R_o_* is the outer radius. The radial strain can be calculated as:

(2) $$\varepsilon_{rr}\left(R_{o}\right)=\exp\left[\frac{p_{o}}{E}\frac{1}{1-a^{2}}\left\{\left(ba^{2}-1\right)\left(1-N_{r\theta }\right)-\left(b-1\right)\left(1+N_{r\theta }\right)\right\}\right]-1$$

where *R*_i_ = *aR_o_* (0 < *a* < 1) and *p*_i_ = *bp*_o_ (*b* > 1);^[^ ^50^ ^]^ *R*_i_ is the inner radius of the tube, *p*_i_ and *p*_o_ are the pressure inside and outside the tube, respectively, and *E* is the elastic modulus, measured as 0.60 MPa (see Section S4, Supporting Information). The calculated peak shift is comparable to the experimental results but smaller. This is because the stiff cholesteric phase near the outer tube surface is less prone to deformation than the inner side (Figure 4e).

Likewise, we can calculate ɛ_θθ_ from ɛ_*rr*_ (see Section S4, Supporting Information), using ɛ_rr_ = −*N*_θr_ɛ_θθ_. *N*_θr_, which depends on pneumatic pressure, decreases from 0.821 to 0.786 when *b* increases from 1.5 to 3, matching the experimental value of 0.77 (see Section S5; Figure S17, Supporting Information). It confirms the enhanced color sensitivity of tube inflation. The tube colors shift from red to blue by pressurizing the tube to 2.3 atm in 0.3 s (Figure 4f), with the peak shifting from 610 to 450 nm in 1.1 s. The restoration is slower, taking 1.2 s to reach 550 nm, 2.2 s to 575 nm, 7.5 s to 600 nm, and 15.6 s to fully return to 610 nm, due to the dissipation of elastic energy within the viscoelastic elastomer. The sensitivity to color changes increases further when inflating a bent tube, as it combines circumferential elongation, axial elongation of the tube wall facing outward, and compression. This is corroborated by the values of *N*_θr_ at the bent curvatures of 0.29, 0.51, and 0.83 mm^−1^, corresponding to 0.99, 1.05, and 1.13, respectively (Figure 4g). The tube, wound around cylinders of varying diameters, has its colors blue‐shifted before inflation due to bending‐induced stress (Figure 3f).^[^ ^15^ ^]^ A larger curvature further blueshifts the initial reflectance peak upon inflation (Figure S18, Supporting Information).

### 2.4 Mechanochromic Displays

**Figure 5:**
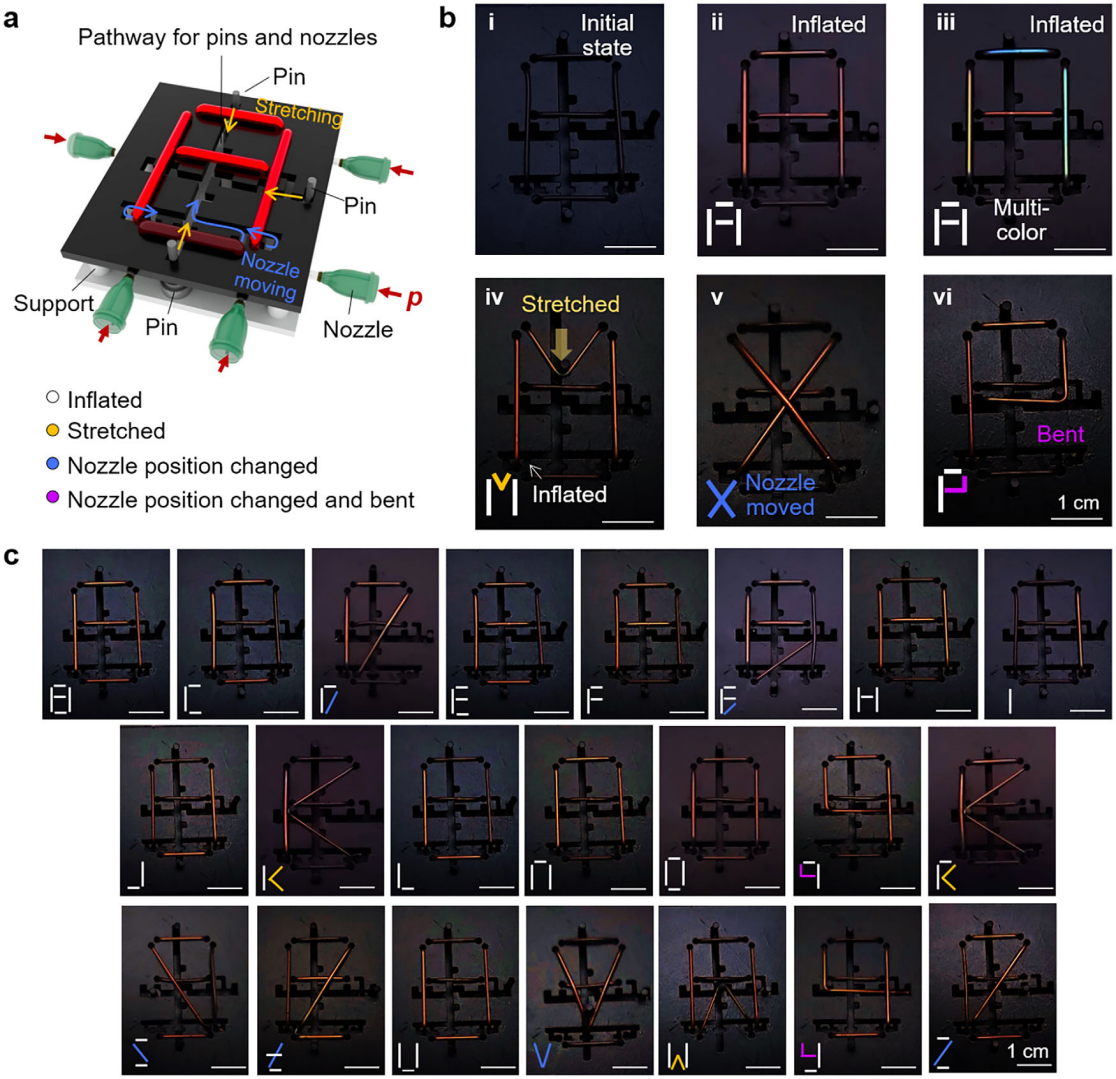
Reconfigurable reflective display showing structural color alphabets. a) Schematic diagram of an alphabet display composed of five CLCE tubes, reconfigurable by the movement of pins and nozzles. b) Photos showing the initial state with NIR colors i), alphabet “A” in a single color ii) and multi‐colors iii) by inflation only, alphabet “M” by inflation and stretching iv), alphabet “X” by nozzle movement and inflation v), and alphabet “P” by bending and inflation of the tubes vi). c) Photos showing the remaining alphabets created by combining four modes of color tuning indicated in a.

To illustrate the unique geometric characteristics of the CLCE tubes and their highly strain sensitive, multi‐mode mechanochromic responses upon extension and inflation, we design and construct an alphabet reflective display system (**Figure** **5**), where the letters or numbers are composed of lines. As tubes are pneumatically inflated, they can be easily repositioned to achieve a different alphabet, while retaining mechanochromic properties. Here, two interlocking substrates are used: the top substrate has perforated pathways for nozzles to move and pins to stretch the tubes (Figure S19, Supporting Information). Initially, the tubes show a reflection peak at 790 nm, displaying blackish colors due to the presence of carbon black nanoparticles and near‐infrared reflection (Figure S20, Supporting Information). The blank display reveals the letter “A” by inflating tubes in red or in five different colors (Figure 5b(i–iii)). The letter “M” is demonstrated by pulling the upper tube down with a pin, inducing blueshift, while inflating two side tubes to match colors (Figure 5b(iv)). The letter “X” is displayed by repositioning the nozzles and inflating the tubes, while the letter “P” forms similarly, with the tube bending over the pin (Figure 5b(v,vi)). These strategies allow us to achieve a reconfigurable reflective display showing all 26 English alphabets using just five CLCE tubes (Figure 5c).

**Figure 6:**
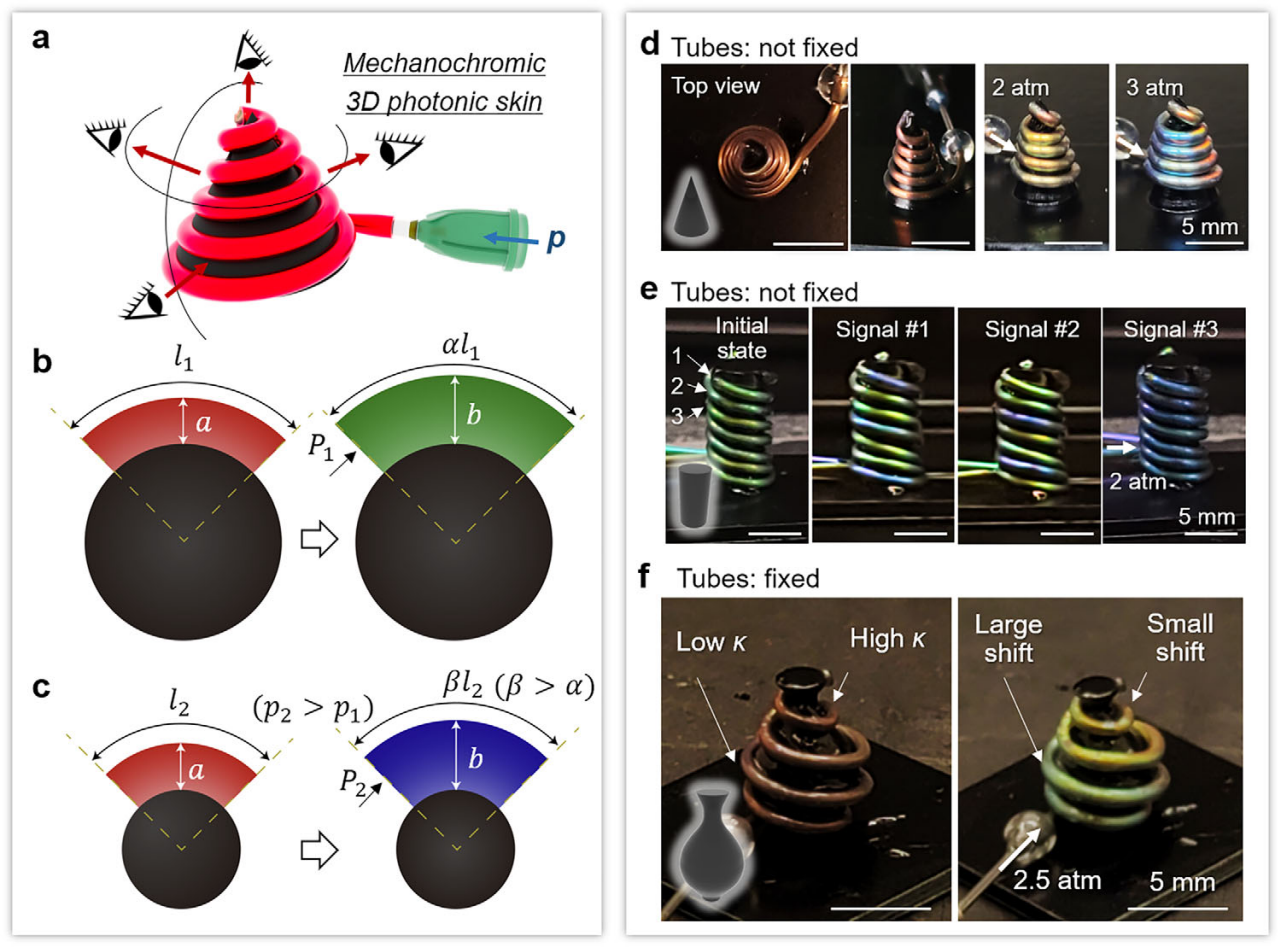
A mechanochromic 3D photonic skin. a) Schematic diagram of a 3D object‐wrapping CLCE tube displaying omnidirectional structural colors. b,c) Schematic illustrations of a bent tube upon inflation at low (b) and high (c) curvatures. d) Photos of a non‐fixed 3D‐printed cone wrapped with a red CLCE tube from the top view and perspective views, respectively, displaying color change upon inflation at different pressures. e) Photos of a non‐fixed 3D‐printed cylinder wrapped with three green photonic tubes (indicated as 1, 2, and 3), displaying multiple signals through selected tube inflation. f) Photos of a 3D‐printed vase wrapped with a red CLCE tube bonded to the vase during tube inflation.

Further, to take advantage of the high sensitivity of the bent tubes, we wrap a 3D object with a CLCE tube, creating a curvature‐dependent 3D photonic skin with omnidirectional SCs (**Figure** **6**a). Higher curvature makes it harder to increase circumferential strain, which accompanies axial elongation (Figure 6b,c). First, a CLCE tube is wound around a cone without fixation, displaying structural color visible from all directions within a hemispherical range (Figure 6d). The color of the entire tube shifts uniformly from red to blue upon inflation, as pressure concentration caused by curvature is alleviated by the tube's axial shift, enabling multi‐directional communication, similar to emergency alarms. Wrapping three identical tubes around a cylinder allows for a display viewable from a 360° angle, where each tube can be individually actuated (Figure 6e). Finally, we wrap a single tube around a vase of gradient curvatures, followed by fixing the tube to prevent it from sliding on the vase. Different colors from the same tube are observed at different tube locations. This is because the tube wrapped at the lower curvature region experiences less pre‐stress, thus exhibiting a larger shift of color upon inflation.

## 3 Conclusions

In conclusion, we have developed a tubular structural color platform at a sub‐millimeter scale, demonstrating multi‐modal, reconfigurable, mechanochromic responses. By injecting air into a highly viscous CLC precursor, a uniform sheath is formed with enhanced cholesteric alignment before gravity and Plateau‐Rayleigh instability take effect. The high molecular anisotropy in the tube sheath, together with the tubular geometry, brings about highly sensitive mechanochromic responses under both extension and inflation. We illustrate the applications of the CLCE tubes in reconfigurable alphabet displays and curvature‐dependent 3D photonic skins. Compared to previous matrix‐driven CLCE systems that rely on an engineered matrix (e.g., stretchable sheet, pixel array, or elastomeric skin) to constrain and drive the CLCE's optical/mechanical response,^[^ ^5^, ^6^, ^29^, ^51^ ^]^ our tubular actuator operates autonomously in free space with multiple modes of actuation, which can be helped by a matrix to enhance display versatility (Table S1, Supporting Information). The study presented here paves the way for programming different modes of structural colors from the same material, making it highly attractive for applications such as soft robotics, displays, sensors, and miniaturized spectrometers.^[^ ^52^ ^]^

## 4 Experimental Section

### Materials

1,4‐Bis‐[4‐(3‐acryloyloxypropyloxy) benzoyloxy]‐2‐methylbenzene (RM257, 98%) was purchased from Henan Wentao Chemical Product Co., Ltd. 4‐cyano‐4′‐pentylbiphenyl (5CB, 98%) was purchased from Ambeed. (3R,3aS,6aS)‐Hexahydrofuro[3,2‐b]furan‐3,6‐diyl bis(4‐(4‐((4‐(acryloyloxy)butoxy)‐carbonyloxy)benzoyloxy)benzoate) (LC756, 82%) was purchased from Chemfish Tokyo. 2,2′‐(Ethylenedioxy) diethanethiol (EDDT, 95%), 1,8‐diazabicyclo[5.4.0]undec‐7‐ene (DBU, 98%), 2,2‐dimethoxy‐2‐phenylacetophenone (DMPA), trimethylolpropane ethoxylate triacrylate (ETPTA, Mn 428), and 2‐hydroxy‐2‐methylpropiophenone (Darocur 1173) were purchased from Sigma–Aldrich. Toluene (Certified ACS) and methylene chloride (DCM, Stabilized/Certified ACS) were purchased from Fisher Scientific. Carbon black (Hiblack 40B2, average size, 23 nm) was purchased from Orion. Polydimethylsiloxane (PDMS, Sylgard 184 Silicone Elastomer Kit) was purchased from Dow Corning. Steel wires with a diameter of 640 µm were purchased from Master Wire Supply. 20‐gauge needles were purchased from Thermo Fisher Scientific. Plastic tubing with a diameter of 1350 µm was purchased from Scientific Commodities, Inc. All chemicals were used without further purification.

### Synthesis of the CLCE Precursors

First, CLC oligomers were synthesized with the molar ratio of acrylate to thiol 0.75 following the literature.^[^ ^31^ ^]^ Briefly, 9.50 g RM257, 0.50 g LC756, 4.03 g EDDT, and 125 mL DCM were mixed in a round bottom flask. 50 µL DBU was added as a catalyst, and then the flask was sealed with an Al foil, stirred at 500 rpm at room temperature. After 14 h, the mixture was poured into a 200 mL flask, then 75 mg of BHT was added as an inhibitor. The flask was kept at 350 K on a hot plate for 18 h to evaporate the solvent. To prepare the CLCE precursor, 1.54 g CLC oligomers, 413 mg RM257, 27 mg LC756, 60 mg 5CB, 80 mg DMPA, and 50 µL toluene were added in a vial. If black samples are needed, 3.0 mg of carbon black was added. The vial was sealed and heated in an oven at 363 K for 10 min, then stirred mechanically with a glass rod until a uniform dispersion was achieved, then heated at 363 K again for 1 h to eliminate the bubbles. After that, the ink was transferred into a 3 mL syringe for use. This recipe gives red CLCE. In the ink mixing step, the amount of RM257 and LC756 can be adjusted to tune color, with the sum of liquid crystal monomers RM257 and LC756 as 440 mg.

### Fabrication of the PDMS Channels

The PDMS channels with diameters of 640 µm, 910 µm, and 1350 µm were molded from a steel wire, a steel needle, and a plastic tube, respectively. The fibrous structures were secured in a plastic Petri dish either by piercing them through the dish or by clipping. PDMS, mixed in a 10:1 ratio of base to curing agent, was poured into the dish to make the PDMS thicker than 1 cm, and degassed for 10 min in a vacuum chamber. After curing at 70 °C for 2 h in a convection oven, the fibrous structures were pulled out from the cured PDMS.

### Fabrication of CLCE Tubes

The CLCE precursor was injected into the PDMS channels and incubated for 2 min to develop coloration. Air was then introduced using a syringe to form an air core, with the injection speed adjusted to the desired rate. Once the air front reaches the other end of the channel, the air was injected from the other side at the same speed. This additional step improves the geometric and color uniformity of the CLCE tubes, as the second air front experiences reduced shear force due to the decreased precursor volume in the channel. After an additional 1 min incubation, the tube was cured under UV light (365 nm, 735 mW/cm^2^, CS2020, Thorlabs) for 1 min. Finally, the CLCE tube was pulled out from the PDMS channel while cutting the PDMS.

### Atomic Force Microscopy Indentation Test

Atomic force microscopy (AFM)‐based indentation tests were conducted by Asylum MFP 3D AFM. The PPP‐CONTR‐50 probe manufactured by NANOSENSORS was selected. The spring constant of the probe cantilever was 0.2 N m^−1^, which was further calibrated using thermal tune method. The tip radius was measured as 80 nm.

### Finite Element Method Simulation

The finite element method (FEM) simulations were performed in Abaqus/Standard (Dassault Systèmes) to simulate the shape change of the stretching of the fiber and tube. The geometries were imported into Abaqus CAE as step files and meshed with solid quadratic tetrahedral elements (C3D10H). A mesh refinement study was applied to verify the accuracy of the mesh. The fiber and tube were modeled as the linear elastic model with Young's Modulus *E* = 0.6 MPa, and Poisson's ratio *υ* = 0.33, respectively. Static simulation was performed as stretching was applied.

### Preparation of Reconfigurable Reflective Display

A digital light processing printer (Sonic Mini 8K, Phrozen) was used to print the substrates and pins from a gray rigid resin (Aqua‐Gray 8K, Phrozen). The 3D models, designed in MAYA (Autodesk) and exported as STL files, were printed and then sonicated in isopropanol for 10 min. The structures were dried in a 60 °C convection oven for 10 min and post‐cured under UV light (270 nm, 1 mW cm^2^, UV‐C LED strip light from Waveform Lighting) for 20 min. To enhance the visibility of structural colors, the printed structures were coated with black spray paint (Satin black, Krylon). One end of the CLCE tubes was sealed with a UV‐curable adhesive (Photobond GB368, Delo), while the other end was connected to nozzles. Both ends were concealed beneath the top substrate, with the glued end secured further with the adhesive underneath the substrate. The end with the nozzle extended at the edge of the gap to connect to a syringe for integration into the pneumatic system. Pins were also installed between two substrates and stood on the bottom substrate, while the thin cylinder protruded out through the perforated regions. These pins traversed through the pathways to either stretch the tube or prevent it from being dragged during the bending process.

### Characterization

The tubes were characterized using an optical microscope (BX61, Olympus). Reflection‐mode imaging was employed to observe their structural colors, while molecular alignment was assessed using a polarizer in the same mode. Tube thicknesses were evaluated either from top‐view images in the transmission mode or from the cross‐sectional images in the reflection mode (Figures S7 and S8, Supporting Information). It was important to note that top‐view images do not accurately represent the true wall thickness due to light diffraction at the interface between materials of different refractive indices. The presence or absence of a PDMS mold during observation alters the light path and thereby affects the apparent thickness. It also alters the refractive index contrast at the outer wall of the tube, thereby causing different light diffraction and shifting the apparent position of the inner wall in the optical image. For direct thickness measurements via the cross‐sectional imaging, the tubes were filled with ETPTA containing 1 wt.% Darocur 1173 and subsequently photo‐crosslinked to preserve their circular cross‐sectional shape during cutting.

## Conflict of Interest

The authors declare no conflict of interest.

## Author Contributions

J.B.K and S.L. contributed equally to this work. J.B.K and S.Y. conceived ideas and designed the research. S.L. developed the CLCE recipe. J.B.K and S.L. fabricated samples, characterized their properties, and demonstrated their applications. S.L. performed theoretical calculations of the mechanics of fiber/tube stretching and tube inflation. J.B.K. tested rheological and interfacial data. K.‐Y.W. performed AFM indentations. Y.C. conducted FEM simulations. S.Y. supervised the project. J.B.K., S.L., and S.Y. wrote the manuscript. All authors participated in discussions and reviewed the manuscript.

## Supporting information



Supporting Information

## Data Availability

The data that support the findings of this study are available in the supplementary material of this article.
