# Supplementary material for: Multi‐Mode Mechanochromic Responses from Cholesteric Liquid Crystal Elastomer Tubes of Uniform Sheath
Source: Adv Mater. 2025 Jun 10;37(34):2504461. doi: 10.1002/adma.202504461 (PMC12392860; doi:10.1002/adma.202504461)
Supplement: Supplementary file 1 — Supporting Information [file ADMA-37-2504461-s001.pdf]

ADVANCED MATERIALS

Supporting Information

for *Adv. Mater.*, DOI 10.1002/adma.202504461

Multi-Mode Mechanochromic Responses from Cholesteric Liquid Crystal Elastomer Tubes of Uniform Sheath

*Jong Bin Kim, Shangsong Li, Kun-Yu Wang, Yinding Chi and Shu Yang**

Supporting Information

Multi-mode Mechanochromic Responses from Cholesteric Liquid Crystal Elastomer Tubes of Uniform Sheath

*Jong Bin Kim, Shangsong Li, Kun-Yu Wang, Yinding Chi, and Shu Yang**

Department of Materials Science and Engineering, University of Pennsylvania, 3231 Walnut Street, Philadelphia, PA 19104, USA

*Corresponding Author: shuyang@seas.upenn.edu

This file includes

- Sections S1 to S5
- Figs. S1 to S20
- Table S1
- References

Section S1. Specular reflection test to confirm the vertical alignment of the CLC phase.

The normal reflection condition follows the Bragg's law, $\lambda = 2dn_{\mathrm{eff}}$, where λ is the reflection peak, d is the interplanar spacing, and n_{eff} is the effective refractive index of the CLCE, which is assumed to be 1.55.[1] A deflated tube exhibiting red structural color shows a normal reflection peak at 758 nm, from which d is calculated to be 244.5 nm. If the CLC phase is tilted at the surface due to shear forces, specular reflections at equal but opposite angles should yield different reflection peaks.[2] The light source and the detector are aligned for specular reflection with both incident and reflected angles set to 45°. The observed reflection peaks are 663 nm and 666 nm from the two opposite directions, supporting the presence of a vertically aligned CLC phase at the tube surface (Figure S4a,b, Supporting Information).

When light impinges obliquely on a structural color sample, reflection peaks can be predicted using the Bragg-Snell's law: $\lambda = 2d\sqrt{n_{eff}^2 - \sin^2\theta}$, where θ is the angle of incidence and reflection. Using the measured λ values and calculated d, the corresponding incident angles θ are computed to be 48.5° and 47.4°, which are consistent with the experimental setup at 45°. After inflating the tube to 2 atm, the same procedure yields reflection peaks of 645 nm under normal incidence and 573 nm and 577 nm under specular reflection from the opposite directions (Figure S4c,d, Supporting Information). The resulting calculated d is 208.1 nm, and the corresponding θ values are 45.4° and 43.8°, again matching the experimental configuration. These calculations validate the structural integrity of the lamellar arrangement at the surface of the CLCE tubes.

Section S2. The stretching models of the CLCE fibers and tubes with cylindrical axial symmetries.

A cylindrical-polar coordinate system, (r, θ, z), is used in all analyses. For a CLCE fiber, a uniaxial strain is applied with other boundaries free. When the tube length is L and the applied stretching pressure on top and bottom of the fiber is p, all surfaces except $z = \pm L/2$ are stress-free, giving the traction vector $\mathbf{t} = (0,0,0)$. At $z = \pm L/2$, the unit normal vector $\mathbf{n} = (0,0,1)$, and $\mathbf{t} = p(0,0,1)$. We calculate the stress by applying these conditions with the boundary condition of $t_i = \sigma_{ji} n_j$, where σ_{ij} is the stress tensor, and i, j correspond to cylindrical coordinates r, θ, z. Also, the boundary condition needs to satisfy the equilibrium condition of $\sigma_{ij,j} + p_i = 0$. Considering the gravity effect of a CLCE tube is negligible compared to the mechanical force applied in stretching or compression, we assume there is no body force as $\mathbf{p} = (0,0,0)$.

Combining all the conditions above, the stress tensor is given by[3]

$$\sigma_{ij} = \begin{bmatrix} 0 & 0 & 0 \\ 0 & 0 & 0 \\ 0 & 0 & p \end{bmatrix} \tag{1}$$

Assuming that the tube is made from an isotropic material with the Poisson's ratio ν and the Young's modulus E, the strain tensor ε_{ij} is[3]

$$\varepsilon_{ij} = \frac{1}{E}\left[(1+\nu)\sigma_{ij} - \nu\delta_{ij}\sigma_{kk}\right] = \begin{bmatrix} -\frac{\nu p}{E} & 0 & 0 \\ 0 & -\frac{\nu p}{E} & 0 \\ 0 & 0 & \frac{p}{E} \end{bmatrix} \tag{2}$$

The expressions for the radial strain ε_{rr}, the circumferential strain $\varepsilon_{\theta\theta}$, and the axial strain ε_{zz} are

$$\varepsilon_{rr} = \varepsilon_{\theta\theta} = -\frac{\nu p}{E} = -\nu\varepsilon_{zz}, \tag{3}$$

$$\varepsilon_{zz} = \frac{p}{E}. \tag{4}$$

Since the radial strain changes the helical pitch of the CLCE directly, it also changes the CLCE color. Given the reflection peak wavelength of CLCE is λ, the change of the peak wavelength is $\Delta\lambda$, the normalized wavelength change $\frac{\Delta\lambda}{\lambda}$ is

$$|\frac{\Delta\lambda}{\lambda}| \leq |\varepsilon_{rr}| = \frac{\nu p}{E}. \quad (5)$$

We conclude that for uniaxial stretching of a CLCE fiber: (1) a tensile pressure induces the normalized wavelength change of $\frac{\nu p}{E}$, corresponding to a magnitude of sensitivity $\frac{\nu}{E}$ under the applied pressure p, and (2) the wavelength change is $\varepsilon_{rr} = -\nu\varepsilon_{zz}$, corresponding to a magnitude of sensitivity ν with applied strain ε_{zz}.

For a CLCE tube with an inner radius R_i, an outer radius R_o, and a length L, a uniaxial strain is applied in the axial direction with other boundaries free. We compare the boundary conditions of tube stretching with the fiber stretching, and there is no difference between them. All surfaces except $z = \pm L/2$ are stress-free. Compared with fiber, the tube has an inner surface, but since it is free with no stress applied, it does not change the stress tensor. Therefore, we still have the same conclusions (1) a tensile pressure p induces wavelength change of $\frac{\nu p}{E}$, and (2) with applied strain ε_{zz}, the wavelength change is $\varepsilon_{rr} = -\nu\varepsilon_{zz}$, which means a magnitude of sensitivity ν with applied strain ε_{zz}. However, in actual experiments, this is not the case. Stretching a CLCE tube shows a higher sensitivity than stretching of CLCE fiber, suggesting that the simple model, as shown above, deviates from the real situation. Detailed discussions on the reason can be found in the main text.

Section S3. Inner air volume change in a tube made from an isotropic material under stretching.

We introduce a linear elastic model to see how the air volume of a tube made from an isotropic material change with strain. We define the true applied axial strain as ε_{zz}, the deformed tube length as L', deformed inner radius as R_i', and deformed outer radius as R_o'. We compute the axial strain ε_{zz} as $\varepsilon_{zz} = \ln\left(\frac{L'}{L}\right)$, thus $L' = Le^{\varepsilon_{zz}}$. Likewise, the radial strain ε_{rr} is $\varepsilon_{rr} = -\nu\varepsilon_{zz}$, thus $R_i' = R_i e^{\varepsilon_{rr}} = R_i e^{-\nu\varepsilon_{zz}}$ and $R_o{}' = R_o e^{\varepsilon_{rr}} = R_o e^{-\nu\varepsilon_{zz}}$. As the initial volume of the air inside the tube V_0 is $V_0 = \pi R_i^2 L$, the volume of air inside the tube after deformation V is $V = \pi(R_i e^{-\nu\varepsilon_{zz}})^2 L e^{\varepsilon_{zz}} = \pi R_i^2 L e^{\varepsilon_{zz}(1-2\nu)}$. Therefore, the change in volume ΔV is[3]

$$\Delta V = V - V_0 = \pi R_i^2 L(e^{\varepsilon_{zz}(1-2\nu)} - 1). \quad (6)$$

As $\varepsilon_{zz} > 0$ during the stretching process and $0 < \nu < 0.5$ for Poisson's ratio ν, $e^{\varepsilon_{zz}(1-2\nu)} - 1 > 0$ and $\Delta V > 0$. The relative volume change can be calculated by $\frac{\Delta V}{V_0}$. Namely, the change in volume in a CLCE tube under stretching is also positive. A higher axial strain will lead to a larger air volume, thus a smaller inner pressure, thus a smaller blue shift. Clearly, this model deviates from experimental results (Figure S15), as it only predicts the initial volume increase but not the volume decrease subsequently.

Section S4. The model of tube inflation.

As the tube length L is much larger than its outer radius R_o, the boundary effect can be neglected during tube inflation. The governing equations for axisymmetric stresses in radial direction σ_{rr}, circumferential direction $\sigma_{\theta\theta}$, and axial direction σ_{zz} are[4]

$$\sigma_{rr} = C_1(1 + 2\ln r) + 2C_2 + C_3\frac{1}{r^2}, \tag{7}$$

$$\sigma_{\theta\theta} = C_1(3 + 2\ln r) + 2C_2 - C_3\frac{1}{r^2}, \tag{8}$$

$$\sigma_{zz} = 4\nu((C_1 + \ln r) + C_2) \tag{9}$$

where $C_1, C_2,$ and C_3 are the constants depending on the boundary conditions. The governing equations for the displacements in radial direction u_r and circumferential direction u_θ are[4]

$$u_r = \frac{1}{E}\left\{C_1 r[(1-\nu)(2lnr - 1) - 2\nu] + 2C_2(1-\nu)r - C_3\frac{1+\nu}{r} + C_4\sin\theta + C_5\cos\theta\right\}, \tag{10}$$

$$u_\theta = \frac{1}{E}[4C_1 r\theta + C_4\cos\theta - C_5\sin\theta + C_6 r] \tag{11}$$

where $C_4, C_5,$ and C_6 are also the constants depending on the boundary conditions. The condition of axisymmetric displacement indicates that there is no dependency on θ, leading to[4]

$$C_1 = C_4 = C_5 = 0, \tag{12}$$

$$\sigma_{rr} = 2C_2 + C_3\frac{1}{r^2}, \tag{13}$$

$$\sigma_{\theta\theta} = 2C_2 - C_3\frac{1}{r^2}, \tag{14}$$

$$\sigma_{zz} = 4\nu(\ln r + C_2), \tag{15}$$

$$u_r = \frac{1}{E}\left\{2C_2(1-\nu)r - C_3\frac{1+\nu}{r}\right\}, \tag{16}$$

$$u_\theta = \frac{1}{E}C_6 r. \tag{17}$$

As the strains in cylindrical coordinate are $\varepsilon_{rr} = \frac{\partial u_r}{\partial r}$ and $\varepsilon_{\theta\theta} = \frac{u_r}{r} + \frac{1}{r}\frac{\partial u_\theta}{\partial \theta}$,[4]

$$\varepsilon_{rr} = \frac{1}{E}\left\{2C_2(1-\nu) + C_3\frac{1+\nu}{r^2}\right\}, \tag{18}$$

$$\varepsilon_{\theta\theta} = \frac{1}{E}\left\{2C_2(1-\nu) - C_3\frac{1+\nu}{r^2}\right\}, \tag{19}$$

and $\varepsilon_{zz} = 0$ as the tube ends are fixed. Now we calculate the solution by enforcing boundary conditions of $t_i = \sigma_{ij}n_j$ $(i,j \in [r,\theta,z]$ when the internal pressure is p_i from air inflation and the external pressure is atmosphere pressure p_o. At the outer diameter R_o, $\sigma_{rr}(R_o) = -p_o$ as $t_0 = (-p_o, 0,0)$ and $n_0 = (1,0,0)$. At the inner diameter R_i, $\sigma_{rr}(R_i) = -p_i$ as $t_i = (p_i, 0,0), n_0 = (-1,0,0)$. Plugging $\sigma_{rr}(R_o)$ and $\sigma_{rr}(R_i)$ into the equation of σ_{rr}, C_2 and C_3 can be stated as $2C_2 = \frac{p_i R_i^2 - p_o R_o^2}{R_o^2 - R_i^2}$ and $C_3 = \frac{R_i^2 R_o^2 (p_o - p_i)}{R^2 - R_i^2}$. To further simplify the equation with parameters, we define a and b by $R_i = aR_o$ $(0 < a < 1)$ and $p_i = bp_o$ $(b > 1)$. Therefore, we get $2C_2 = \frac{(ba^2-1)p_o}{1-a^2}$ and $C_3 = \frac{a^2(1-b)p_o R_o^2}{1-a^2}$.[4]

Since the CLCE lamellar structure is on the outer surface, the normalized wavelength change $\frac{\Delta\lambda}{\lambda}$ should be dominated by the radial strain at the outer surface $\varepsilon_{rr}(R_o)$, which can be stated as[4]

$$\frac{\Delta\lambda}{\lambda} \approx \varepsilon_{rr}(R_o) = \frac{p_0}{E}\frac{1}{1-a^2}\{(ba^2-1)(1-\nu) - (b-1)(1+\nu)\}, \tag{20}$$

$$\varepsilon_{\theta\theta}(R_o) = \frac{p_0}{E}\frac{1}{1-a^2}\{(ba^2-1)(1-\nu) + (b-1)(1+\nu)\}. \tag{21}$$

In order to study the change of wavelength with an applied pressure, we take b as the variable, and other parameters are substituted by experimental values as $a = 0.55, R_i = 0.6R$, $\nu = 0.41$, $E = 0.60$ MPa, $p_0 = 0.101$ MPa. $\frac{\Delta\lambda}{\lambda}$ at a small strain is $\frac{\Delta\lambda}{\lambda}(b) \approx -0.297b + 0.198$. Considering the actual strain is not very small, we note that ε_{rr} is true strain, but $\frac{\Delta\lambda}{\lambda}$ relates to engineering strain following $\varepsilon_{rr} = \ln(1 + \frac{\Delta\lambda}{\lambda})$, which gives better approximation at large strain as[3]

$$\frac{\Delta\lambda}{\lambda}(b) = \exp\left(-0.297b + 0.198\right) - 1 \tag{22}$$

when no extra pressure is applied, b=1, $\frac{\Delta\lambda}{\lambda} = -0.094$, which is the intrinsic compression from atmosphere pressure. This background peak shift should be deducted when calculating the actual peak shift with enhanced internal pressure. Considering this factor, we modify the functions as

$$\frac{\Delta\lambda}{\lambda}(b) = \exp\left(-0.297b + 0.198\right) - 0.906 \tag{23}$$

We compare this theoretical calculation with experimental results (Fig. 4e), and they match well with each other. At the same time, we can see how the angular strain (here as the true strain) changes with applied pressure,

$$\varepsilon_{\theta\theta}(b) = \exp(0.383b - 0.482) - 1 \tag{24}$$

We again note that $\varepsilon_{\theta\theta}(b) = -0.094$ when b = 1, and this baseline needs to be corrected. Therefore, we deduct this baseline strain into the functions above

$$\varepsilon_{\theta\theta}(b) = \exp(0.383b - 0.482) - 0.906 \tag{25}$$

Clearly, the angular strain increases with the applied pressure.

Section S5. The Poission's ratio upon inflation of a tube.

When inflating a tube, the applied strain $\varepsilon_{\theta\theta}$ can be converted to ε_{rr}, causing wavelength change. Therefore, if we want to describe color change sensitivity by the applied strain, we take the ratio of $-\varepsilon_{rr}$ to $\varepsilon_{\theta\theta}$. Note that this is not a Poisson's ratio, since the *z* direction is not free but fixed. However, for better understanding of the color change sensitivity to the applied strain, we coin it as the confined Poisson's ratio with a different notation $N_{z\theta}$, which is expressed as $N_{z\theta} = -\frac{\varepsilon_{rr}(R_o)}{\varepsilon_{\theta\theta}(R_o)}$. Plug in the equations of ε_{rr} and $\varepsilon_{\theta\theta}$, we have the function $\mathrm{N}_{z\theta}(b)$ below[4]

$$\mathrm{N}_{z\theta}(b) = -\frac{\frac{p_0}{E}\frac{1}{1-a^2}\{(ba^2-1)(1-v)-(b-1)(1+v)\}+\varepsilon_1}{\frac{p_0}{E}\frac{1}{1-a^2}\{(ba^2-1)(1-v)+(b-1)(1+v)\}+\varepsilon_2} \quad (26)$$

where $a = 0.55$, $v = 0.41$, $E = 0.60$ MPa, $p_0 = 0.101$ MPa. ε_1 and ε_2 are the minor adjustments from the background peak shift as mentioned in Section S4. Therefore, the graph of the function $N_{z\theta}(b)$ can be plotted quantitatively (Figure S17). The function diverges at *b* = 1, but quickly becomes stable for *b* > 1.1, and decreases very slowly as *b* further increases, eventually converges at $N_{z\theta} = 0.775$. In actual experiments, the loading is usually in the range of *b* = 1.5 to *b* = 3, where the $N_{z\theta}$ decreases very slowly from 0.821 to 0.786, behaving as a nearly constant value. This aligns with experimental results, which give the value of 0.77 (Fig. 4d).

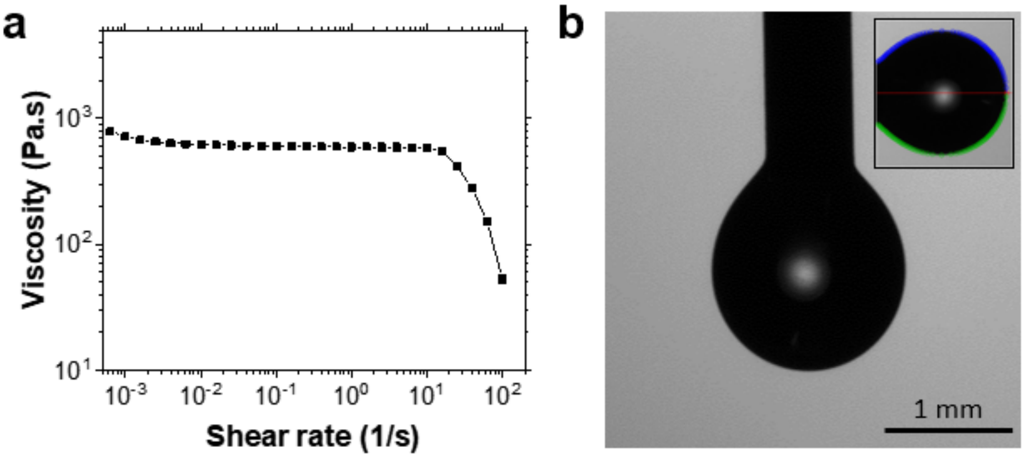

Figure S1. Rheological and interfacial data of the CLCE precursor. a) The viscosity-shear rate relationship. b) Projection image of a pendant drop of the CLCE precursor in the air used for the measurement of its surface tension. The inset shows the computational tracking of the droplet shape.

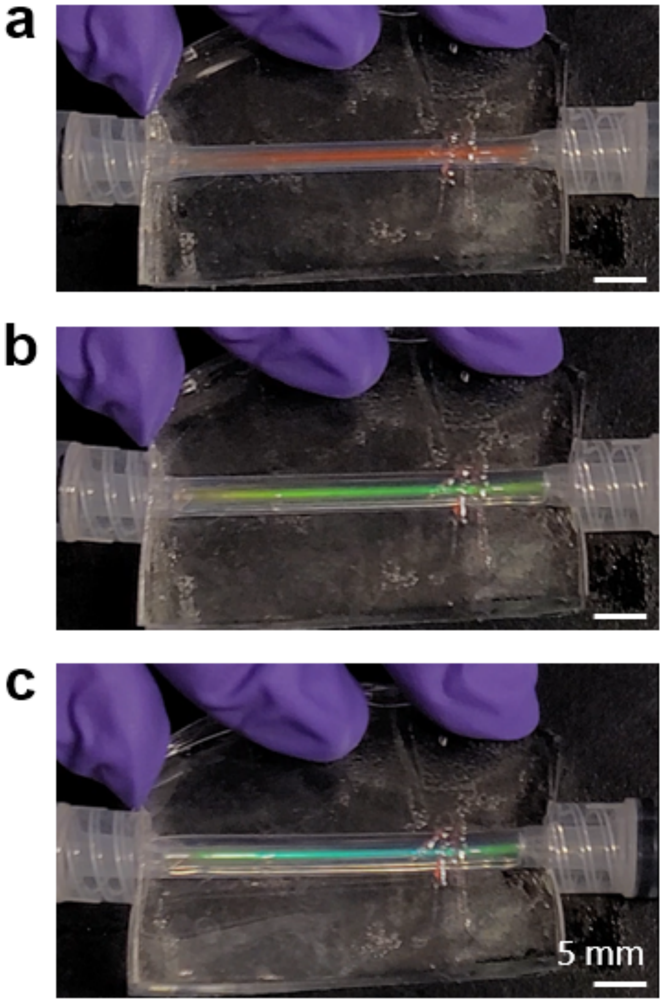

Figure S2. Color change of the CLCE precursor with an air core in the PDMS microchannel with the diameter of 3.2 mm under different pneumatic pressures before curing. a–c) Photos of the CLCE precursor under the pneumatic pressure of 1 atm (a), 3 atm (b), and 5 atm (c), respectively.

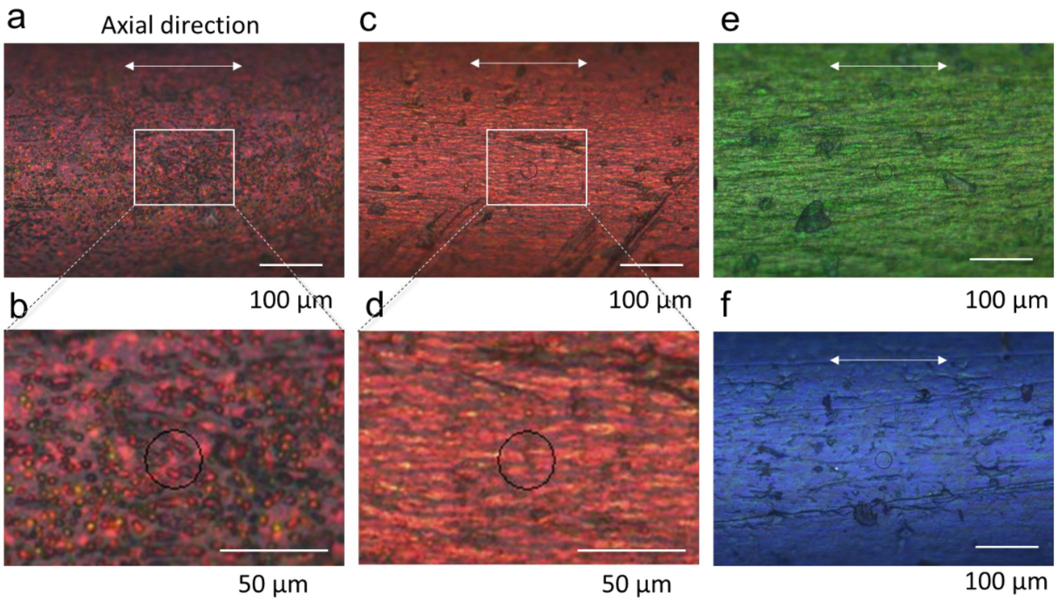

Figure S3. Optical microscopy (OM) images of the surface of a fiber and a tube. a,b) The fiber surface (a) and its magnified view (b). The arrow indicates the axial direction of the fiber. c–f) OM images of the surface of red (c), green (e), and blue (f) tubes, along with a magnified view of the red tube (d).

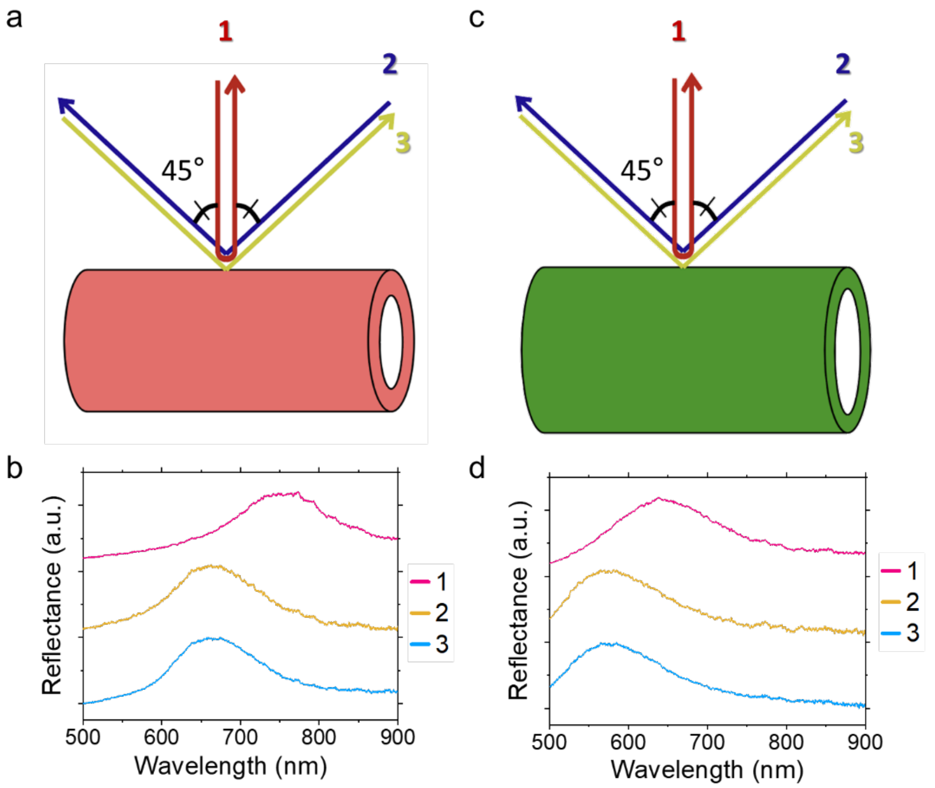

Figure S4. Spectral measurements of the tubes in the normal incidence and specular reflection modes. a,b) Schematic of the experimental setup for measuring the deflated CLCE tubes (a) and the corresponding reflectance spectra (b). The angle of incidence and reflection in the specular reflection is 45°. c,d) Same as a and b, respectively, for the inflated tube.

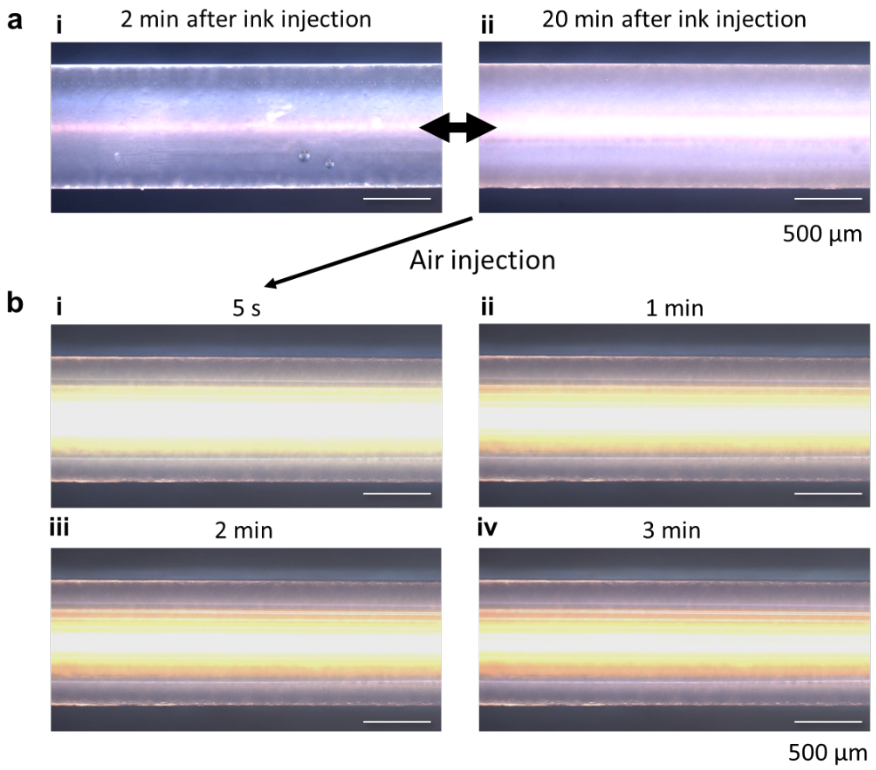

Figure S5. Color development after full coloration in a fibrous geometry and subsequent air injection. a) POM images of the fibrous geometry of the precursor 2 min (i) and 20 min (ii) after filling the CLCE precursor into the PDMS channel. b) Time-lapse POM images taken at various intervals after injecting air, which is performed 2 min after the precursor fills the channel.

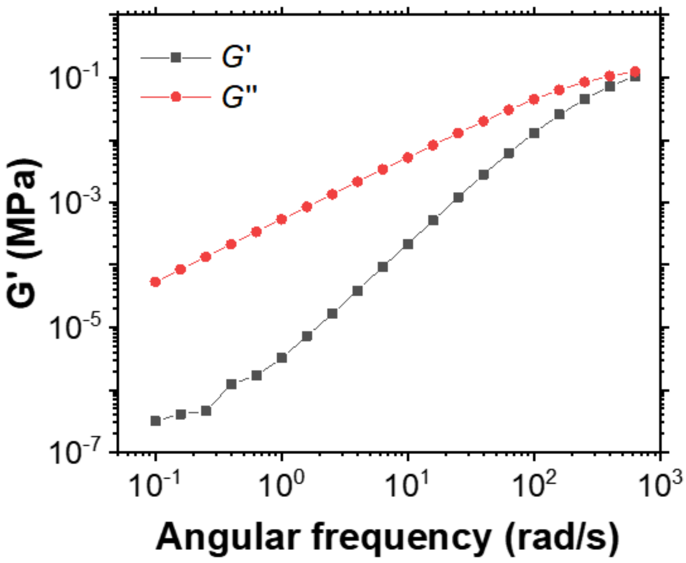

Figure S6. Oscillatory data of the CLCE precursor as a function of the angular frequencies.

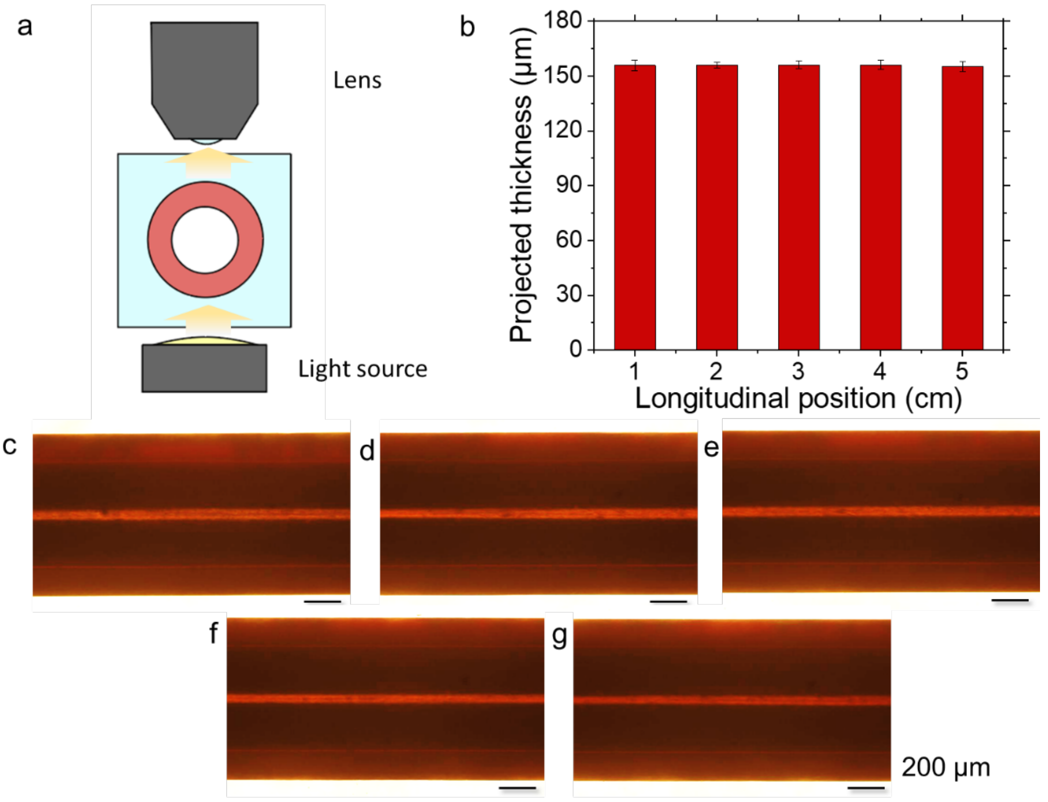

Figure S7. Axial uniformity of the tube thickness. a) Schematic of the optical microscope setup to characterize the axial tube thickness. b) Thickness values measured along longitudinal positions at 1 cm intervals (n = 5). c–e) Top-view OM images corresponding to the longitudinal positions of 1 cm (c), 2 cm (d), 3 cm (e), 4 cm (f), and 5 cm (g) shown in b), respectively. The scale bar shown in (g) is applicable to (c-f).

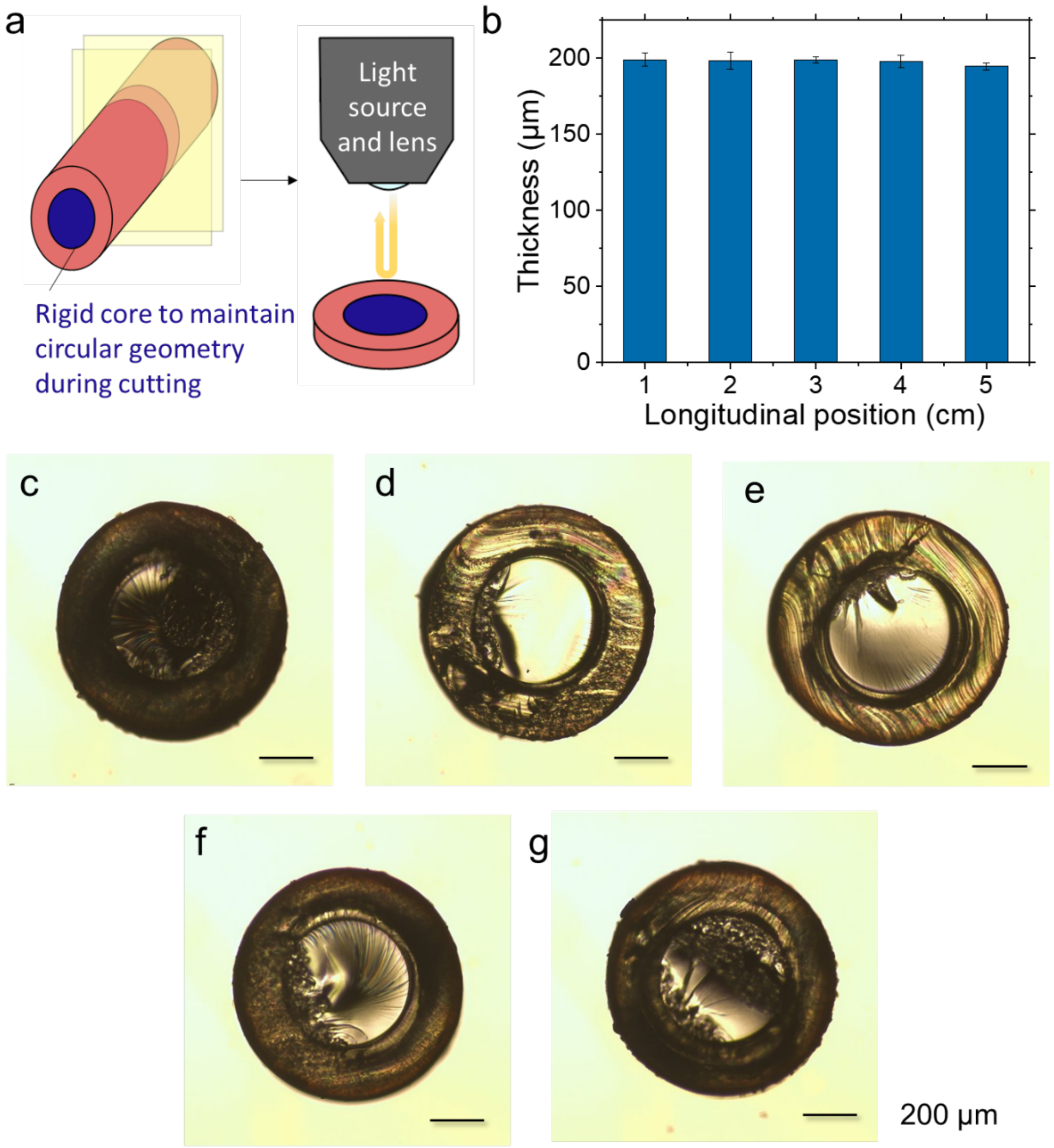

Figure S8. Azimuthal uniformity of the tube thickness. a) Schematic of the sample preparation and the optical microscope setup. b) Thickness values measured along the longitudinal positions at 1 cm intervals (n = 5). c–e) Top-view OM images corresponding to the longitudinal positions of 1 cm (c), 2 cm (d), 3 cm (e), 4 cm (f), and 5 cm (g) shown in b), respectively.

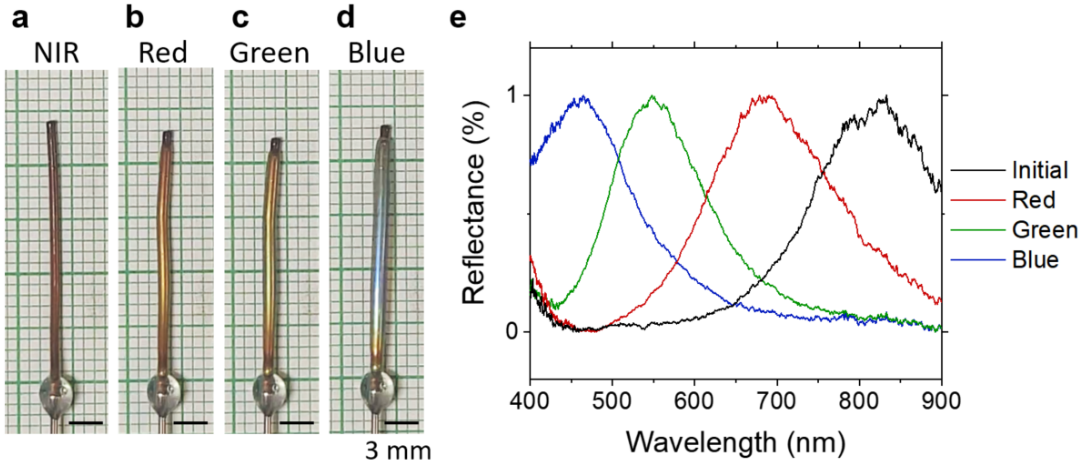

Figure S9. Length and color changes of a CLCE tube upon inflation at different pressures. a–d) Photos of the tubes being inflated from near-infrared at 1 atm (a), to red at 1.90 atm (b), to green at 2.78 atm (c), and to blue color at 3.47 atm (d). e) Reflectance spectra of the four states in a–d.

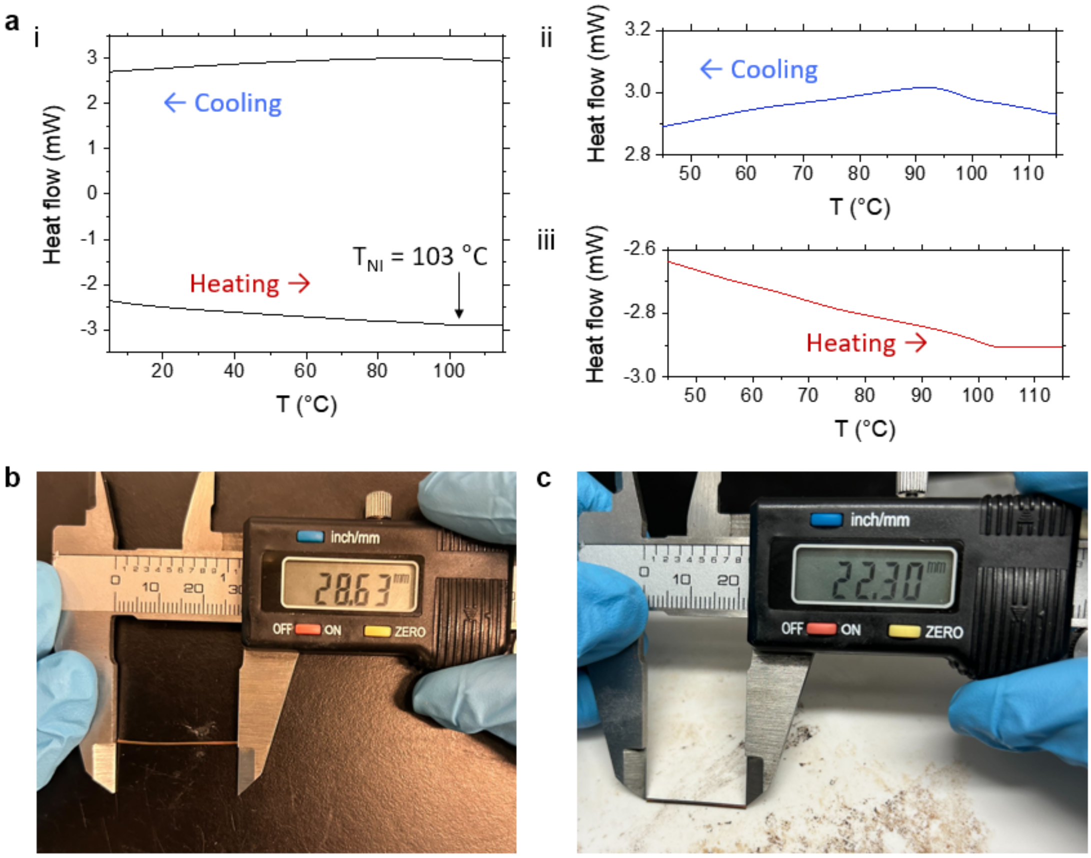

Figure S10. Presence of the pseudo-nematic phase in the CLCE tubes. a) Differential scanning calorimetry analysis (i) showing the nematic-isotropic transition temperature (T_{NI}), with magnified views of each cooling (ii) and heating (iii) cycle. b,c) Axial shrinkage of a CLCE tube induced by temperature shown by the CLCE tubes at room temperature (b) and on an 80 °C hot plate (c).

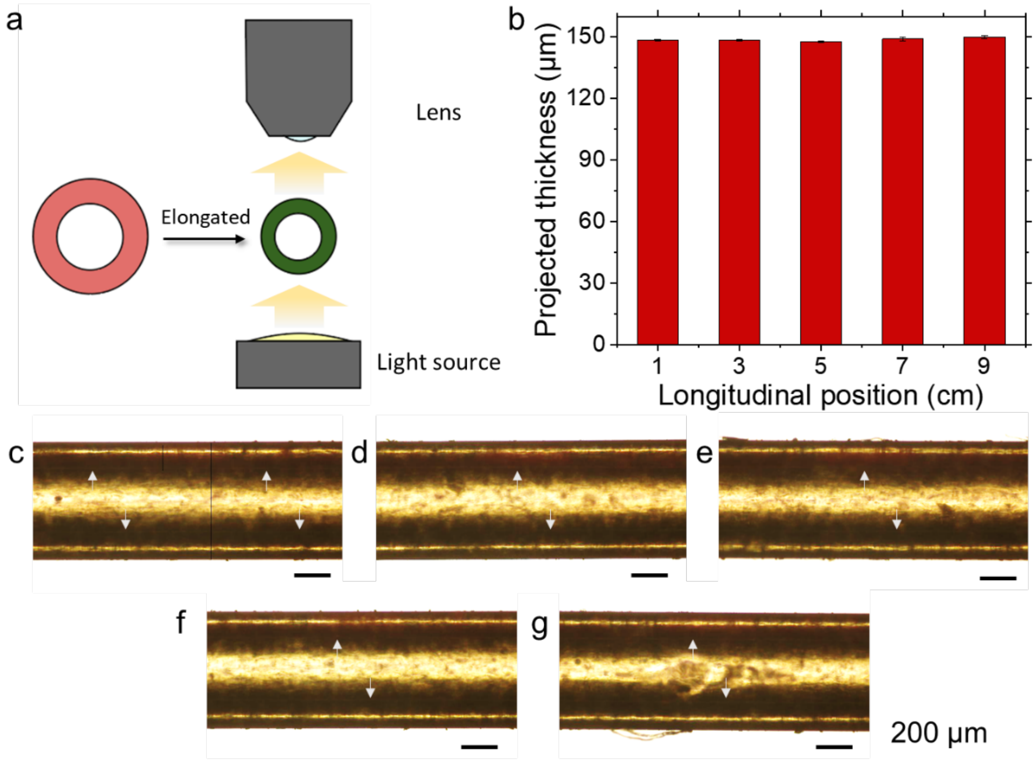

Figure S11. Thickness uniformity upon tube stretching. a) Schematic of the tube cross-section stretched to 200% and the optical microscope setup to observe the change of thickness. b) Thickness values measured at longitudinal positions at 2 cm intervals (*n* = 5). c–e) Top-view OM images corresponding to the longitudinal positions of 1 cm (c), 3 cm (d), 5 cm (e), 7 cm (f), and 9 cm (g) shown in b), respectively. The arrows indicate the positions of the projected boundaries of the inner tube wall. The scale bar shown in (g) is applicable to (c-f).

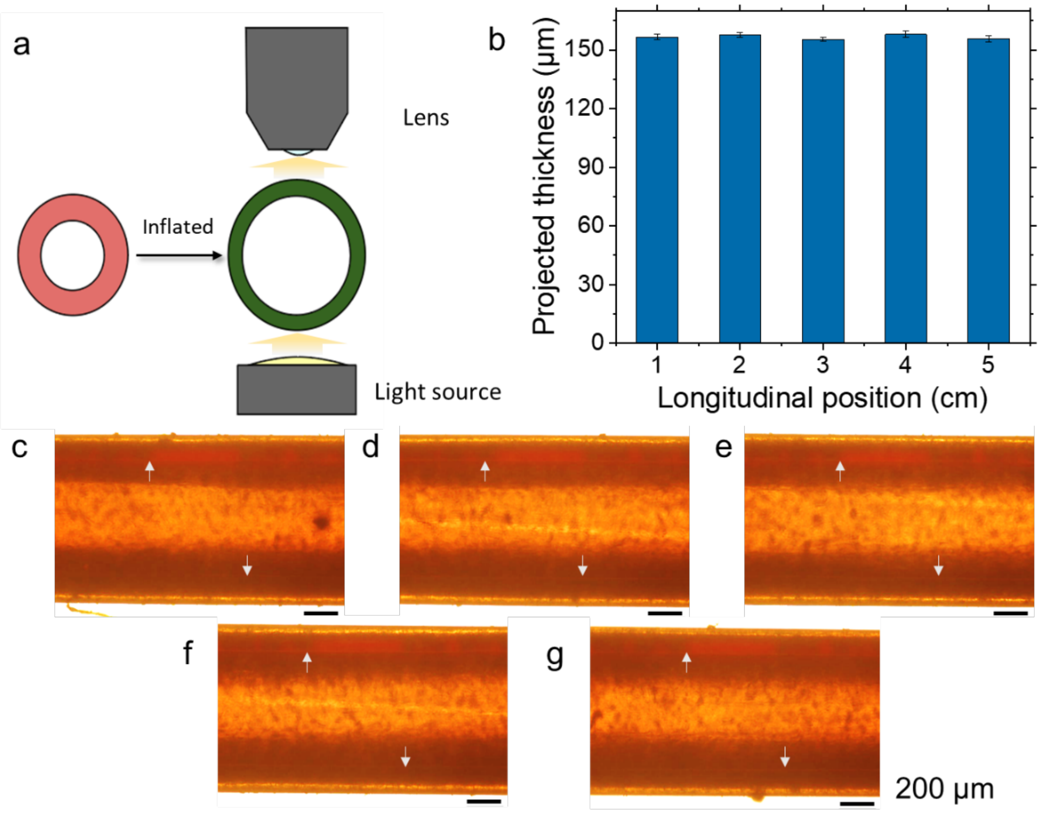

Figure S12. Thickness uniformity upon tube stretching. a) Schematic of the tube cross-section inflated to 2 atm and the optical microscope setup. b) Thickness values measured at longitudinal positions at 1 cm intervals ($n = 5$). c–e) Top-view OM images corresponding to the longitudinal positions of 1 cm (c), 2 cm (d), 3 cm (e), 4 cm (f), and 5 cm (g) shown in b), respectively. The scale bar shown in (g) is applicable to (c-f). The white arrows indicate the positions of the projected boundaries of the inner tube wall.

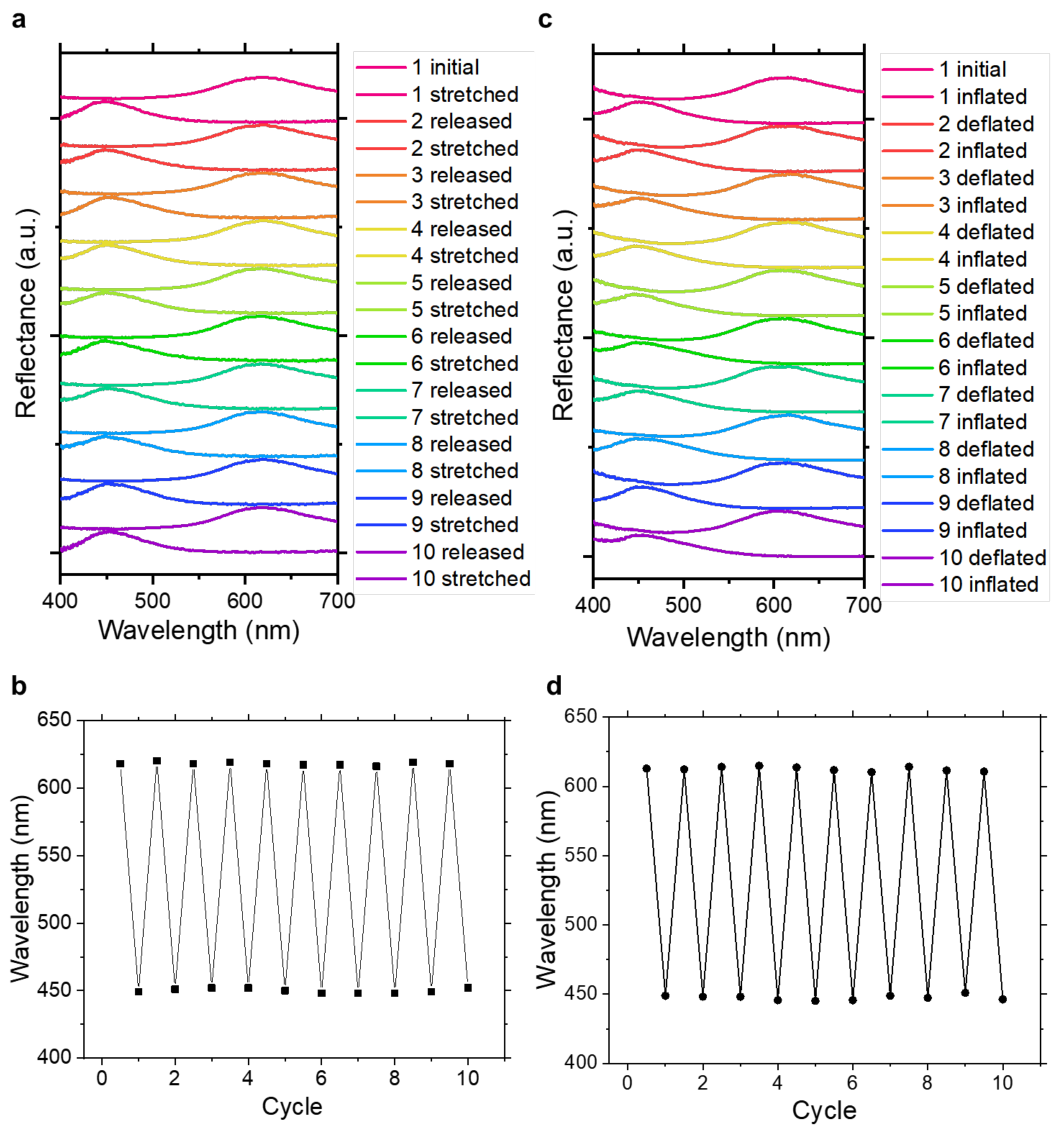

Figure S13. Repeatability of tube color changes upon reversible stretching. a,b) Stacked reflectance spectra of the stretched and released states from 10 cycles with a 220% strain (a), and the corresponding peak wavelengths measured for each state (b). c,d) Same as a and b, respectively, for the inflated and deflated states with pneumatic pressure of 2 atm.

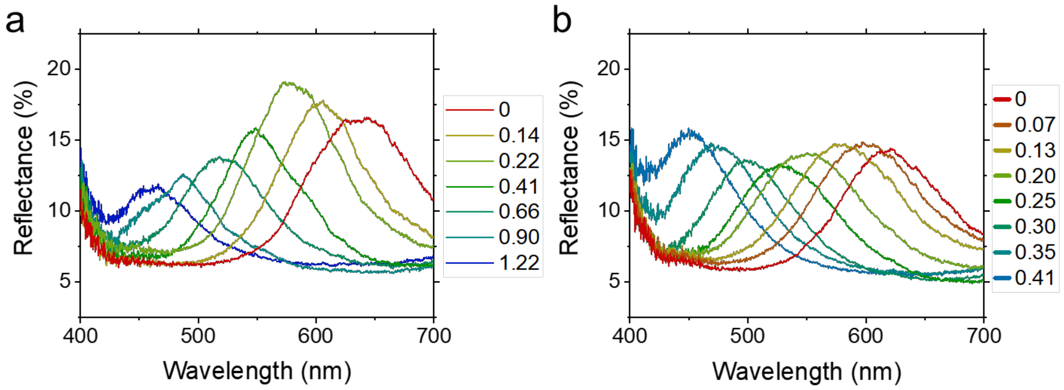

Figure S14. Reflectance spectra of the CLCE tubes upon stretching (a) and inflation (b) with absolute reflectance values.

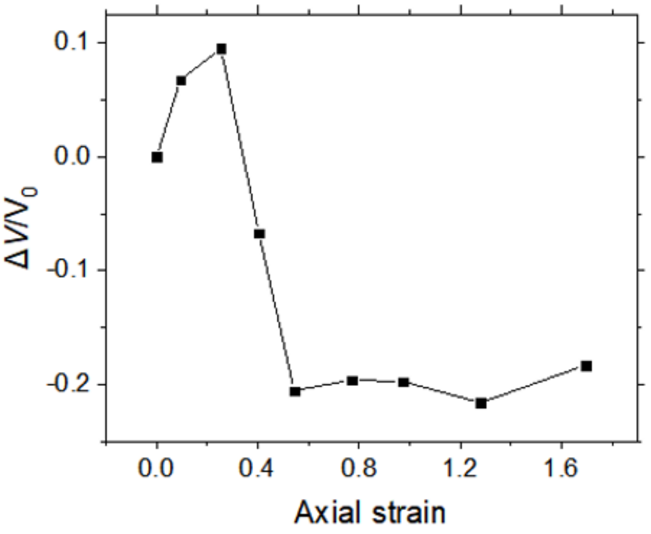

Figure S15. Relative inner volume change $\frac{\Delta V}{V_0}$ of a CLCE tube upon stretching. The inner volume V is measured by observing the inner wall diameter of the tube while stretching the tube.

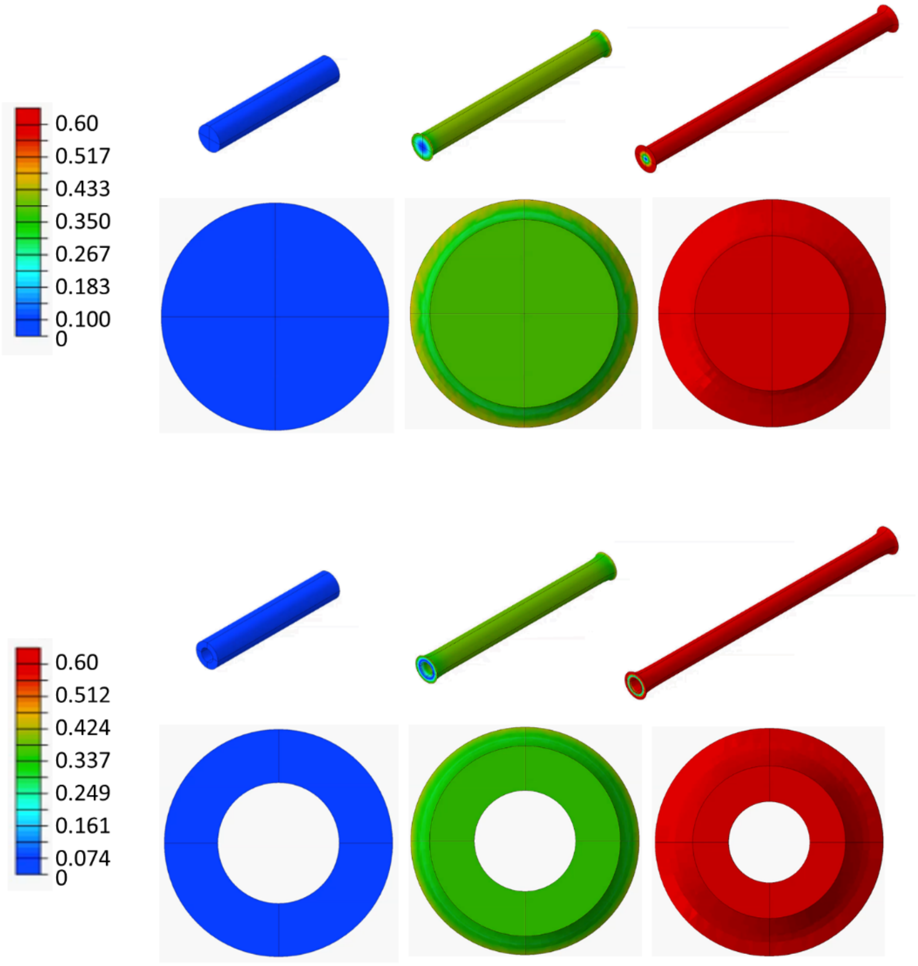

Figure S16. Finite element method (FEM) simulations showing stretching of a fiber and a tube made from an isotropic material. The perspective view (upper row) and the cross-sectional view (lower row) when stretching the tube, showing max principal strains under tension with colors.

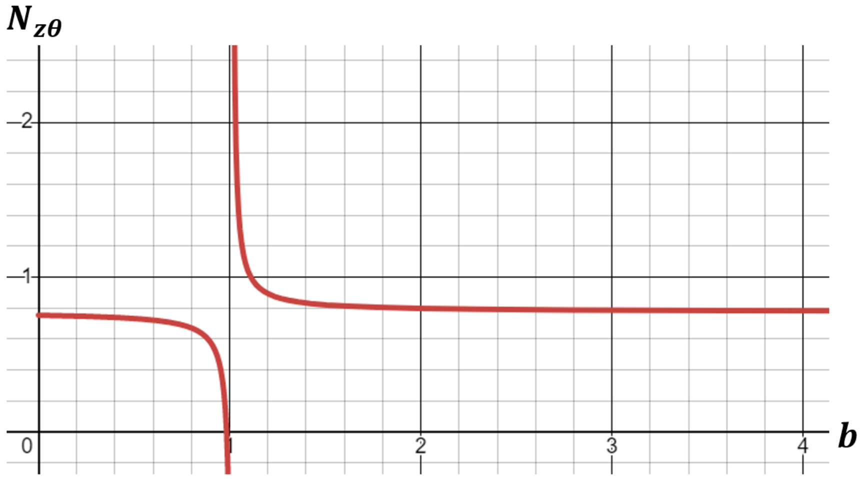

Figure S17. The relationship between geometrically confined Poisson's ratio $N_{z\theta}(b)$, and b from $p_i = bp_o$ $(b > 1)$.

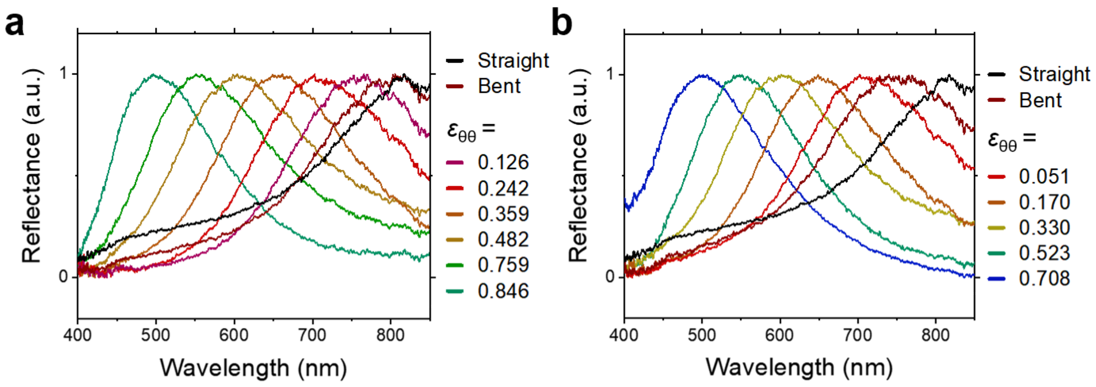

Figure S18. Bent CLCE tubes upon inflation. a,b) Reflectance spectra of the tubes at the curvature of 0.29 mm^{-1} (a) and 0.83 mm^{-1} (b).

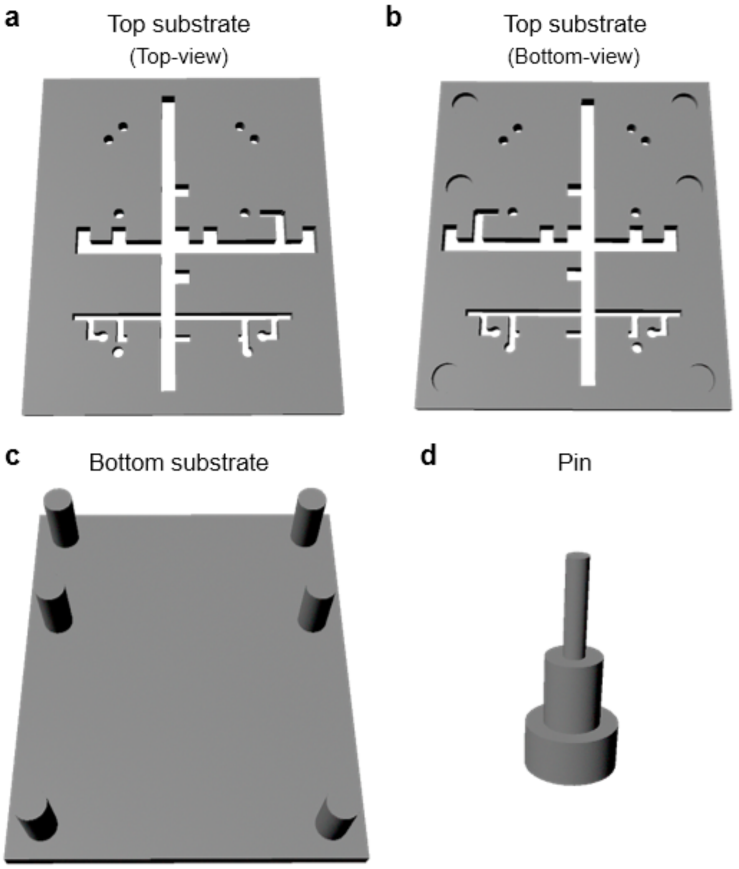

Figure S19. 3D printed models of substrates and pins for a reconfigurable reflective display. a,b) Schematic diagrams of the top- and bottom- views of the top substrate, showing pathways perforated for nozzle and pin movement. c) Schematic diagram of the bottom substrate with posts that can be interlocked with the recessed areas shown in b. d) Schematic diagram of a pin that stretches the CLCE tube as it moves through the perforated pathways.

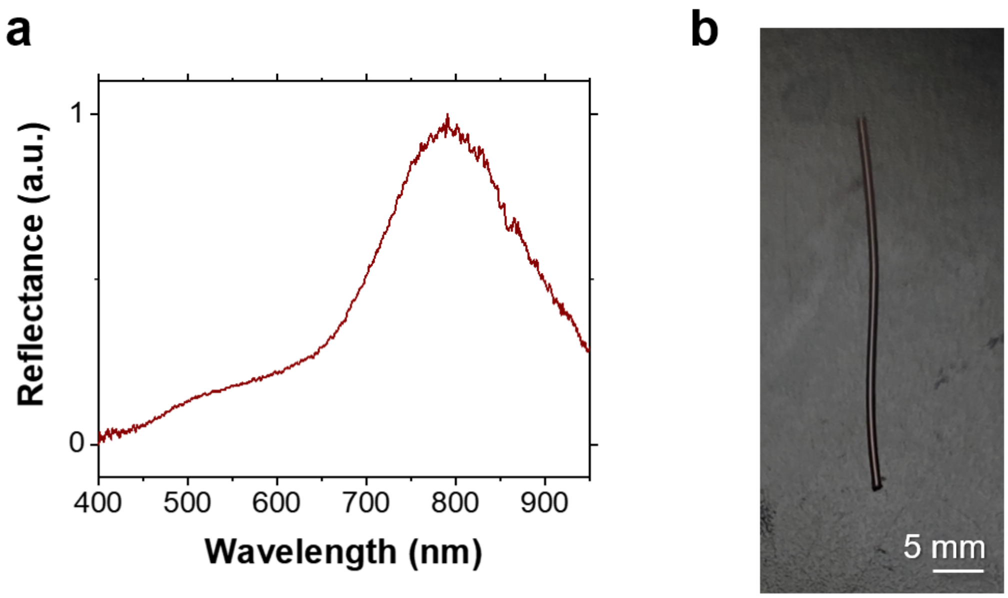

Figure S20. A CLCE tube with color in the near-infrared (NIR). a) Reflectance spectrum of a NIR peak from the tube. b) Photo of the tube.

Table S1. Comparison of the CLCE tubes with previous matrix-driven CLCE systems.

**Aspect**	**This work**	**Nam *et al.*[5] & Shin *et al.*[6]**	**Kim *et al.*[7]**	**Geng *et al.*[8]**
Geometry	Hollow tube	Film on a stretchable electrode	Film on a pneumatic micro-chamber	Fiber
Necessity of a substrate for color change	No<br>(Pneumatic actuation)	Yes	Yes (Pneumatic actuation, but necessitates a pneumatic chamber with cavities)	No<br>(Stretching of fiber)
Actuation mode	1. Axial stretching<br>2. Inflation<br>3. Inflation while bent	Biaxial stretching only	Pneumatic inflation of each cell	Uniaxial stretching only
Color tunability	Broadband<br>(800 nm to 450 nm)	Red to green under the electric field (660 nm to 550 nm)	Broadband<br>(800 nm to 350 nm)	Broadband<br>(625 nm to 470 nm)
Reconfigurability	Tubes can be repositioned, stretched, or bent while being inflatable	Fixed once patterned.	Fixed discrete inflatable pixels	Fixed once fibers are woven

References

[1] J. A. Sol, H. Sentjens, L. Yang, N. Grossiord, A. P. Schenning, M. G. Debije, *Adv. Mater.* **2021**, *33*, 2103309.

[2] J. Choi, Y. Choi, J. H. Lee, M. C. Kim, S. Park, K. Hyun, K. M. Lee, T. H. Yoon, S. k. Ahn, *Adv. Funct. Mater.* **2024**, *34*, 2310658.

[3] A. P. Boresi, R. J. Schmidt, *Advanced Mechanics of Materials*, John Wiley & Sons, Somerset, NJ, **2003**.

[4] V. Vullo, *Circular Cylinders and Pressure Vessels*, Springer International Publishing, Cham, Switzerland, **2013**.

[5] S. Nam, D. Wang, C. Kwon, S. H. Han, S. S. Choi, *Adv. Mater.* **2023**, *35*, 2302456.

[6] J. H. Shin, S. Nam, S. H. Han, J. Y. Park, C. Kwon, S. S. Choi, *Adv. Mater. Technol.* **2023**, *8*, 2301322.

[7] S.-U. Kim, Y.-J. Lee, J. Liu, D. S. Kim, H. Wang, S. Yang, *Nat. Mater.* **2022**, *21*, 41-46.

[8] Y. Geng, R. Kizhakidathazhath, J. P. Lagerwall, *Nat. Mater.* **2022**, *21*, 1441-1447.
